# Objective demonstration and quantitation of musical learning in older adult novices across a 12-month online study

**DOI:** 10.1371/journal.pone.0320055

**Published:** 2025-04-07

**Authors:** Anthony Chmiel, Roger T. Dean, Catherine J. Stevens, Jennifer MacRitchie

**Affiliations:** The MARCS Institute for Brain, Behaviour and Development, Western Sydney University, New South Wales, Australia; University of Western Ontario, CANADA

## Abstract

This work aimed to objectively (mainly computationally) measure the extent to which 68 older adult novices developed specific musical abilities. The participants learned aural and keyboard performance skills in a 12-month online course with an expert piano teacher, spending six months each on a digital piano keyboard and an iPad virtual piano. Within each 6 months, 3 were devoted successively to each of melodic replication and improvisation. Teaching sought correctness of pitches/sequences (for replication), and introduction of systematic diversity thereof (for improvisation). We measured aural perception using melody detection and beat alignment tests; and replication and improvisation learning using computational measures of MIDI-recordings. Bayesian modelling showed that melody detection, replication and improvisation were learned successfully and seemingly progressively, while beat detection, and rhythmic precision in replication, which were not our focus, were not. These skills were retained over a 6-month follow-up period. Improvisation teaching was the bigger predictor of melody detection, and replication teaching of replication performance. Potential applications for these findings in learning contexts are discussed.

## Introduction

Community and health practitioners are increasingly aware of the potential for older adult novices embarking on music instrument learning (i.e. active music-making), particularly for wellbeing [1–3], as well as general cognition [4–6]. By general cognition, we mean mental processes that are not solely intrinsic to the kinds of musical processes we train, having other or broader applicability. Opportunities to learn a musical instrument occur in formal programs or individual music lessons, or informally through technology-mediated access using social media or dedicated apps, such as *Youtube* or *Yousician* [7]. As an intervention to support wellbeing at any life-stage, music seems particularly well-suited to ensure high adherence to a program: since it is individually and emotionally relevant, and offers opportunities for physical movement and development of skill [8].

However, in reports where music instrument learning is used as a wellbeing intervention, there is little focus on how the older adult develops musically, despite the importance of this to both student and educators: most studies lack any measures of musical learning, and the exceptions generally mostly involve subjective assessments (where expert or inexpert listeners give an opinion of the output, and a consensus mark is derived). Occasionally, very limited precise test measures have been used, but often measures are restricted to aural abilities. Additionally, such learning interventions are usually brief (≤ 6 months). We aimed to lengthen this intervention period (12 months, plus a 6 month follow-up period) and broaden and objectify the musical measures.

To take a notable example: one study taught an instrument (the piano) for 12 months (participants aged 62–78), and focused on brain functional connectivity [9]. It measured general sophistication from the Goldsmiths Musical Sophistication Index battery [10] at outset only, and followed a MIDI-based motor-timing precision measure during learning. For this, the scale notes CDEFGFEDC repeated 3 times were to be played isochronically with right-hand fingers 1–5 (two handed playing was taught) and the variability of the timing was the precision criterion. Participants improved in this parameter, although it is a motor synchronisation rather than musical learning measure. A recent detailed review [11] concludes that ‘the evidence that music training causes non-musical benefits is weak or nonexistent’, and discusses musical training in terms of some quantified ‘listening abilities’, but brings forward no evidence on assessment of musical learning per se. In contrast, here we provide clear objective evidence of musical learning, to be applied in subsequent papers to our analysis of broader cognitive and motor changes concomitant with such training.

The reasons for an older adult student to take up instrument learning are reported as health-related, socially-motivated, or more music-specific [2,12–14]. Despite the value for older adult students in being able to assess their own musical outcomes [15], the typical focus in such learning is on the possible wellbeing benefits of musical participation and the idea of active ageing [16]. Across all age groups, but particularly with older adults, few studies have reported on quantitative improvements in musical skill [15] despite Laes’ [17] recommendation that investigation of pedagogically meaningful outcomes should be given attention. As well as exploring how musical learning develops over time for the present cohort, we believe it important to demonstrate to older adults and their supporters the extent to which learning (and potentially, creativity) can still be achieved, to combat external and internalised stigma [17] and difficulties that participants often have in believing they could develop a ‘musician’ identity [18]. We briefly elaborate and discuss creativity in the final section of the paper.

To better understand older adult musical learning, we delivered a 12-month music instrument instruction course to 68 cognitively intact older adults as fortnightly group lessons. We dubbed the course the ‘Active Minds Music Ensemble’ (AMME). While originally designed as in-person group learning, our program almost immediately transitioned online due to COVID-19, and the learners’ experiences are previously discussed [19]. The music lessons were co-designed and delivered throughout by Patrick O’Donnell (B.Mus.Ed., ATCL (Perf), Teachers Cert., A.Mus.A (Musicianship**):** a long-standing member of the NSW Music Teachers’ Association) as part of the research team. This paper assesses how these older adult novices enhanced their music aural perception, and their instrument performance abilities, by objective computational means. We hoped that a composite musical attainment measure (i.e. learning) from the present work might be suitable to use as a predictor in later models of cognitive and motor attainment, potentially replacing the extent of training per se in them. For this purpose, cognitive, motor and wellbeing measures were also taken, and will be assessed in future publications given the musical learning demonstrated here. We make no comparative claims about the relative efficacy of music learning versus, say, writing or physical exercise.

### Designing music learning for older adult students

The choice of how to deliver music instrument lessons might ideally be personalised together with the older learner, taking into account what type of material they would like to learn (playing music from lead lines, improvisation, etc.) [13,15]. Experimentally, instrument teaching programs (predominantly piano-based and short-term) have assessed older adults over time. Instrument training has comprised learning how to reproduce melodies either through the use of traditional staff notation [6,20] or in alternative notations such as FigureNotes [4]. Noting the high memory and cognitive demands that learning to read traditional staff notation brings [13], as well as the difficulties older adults may have from visual processing [15], our program opted for aural (by-ear) training. This is known to lead to improved early-stage development over notation-based approaches, as assessed by performing rehearsed music, sight-reading, playing from memory, playing by ear and improvisation [21, 22].

Having chosen aural training, we considered the desirable relative emphasis on melodic pitch versus rhythmic sequencing. Much traditional Western music uses very limited rhythmic diversity (see also discussion section), and we considered it more transparent to a novice whether a played pitch sequence reproduces an exemplar, than whether a rhythmic structure, potentially at a different tempo, does. We wished to use keyboards (of two kinds, with purposely different physical demands, and affordable for us to provide), and not for example, drumpads, as the attractive training vehicle. Taking these factors together, we concluded that emphasising pitch more than rhythm was entirely reasonable, consistent in some ways with traditional solfeggio systems and their teaching.

The overt emphasis in our teaching was on aural identification of pitch/pitch sequence, and interpreting and then performing and varying these. One cannot create or recreate a recognisable melody without pitch accuracy, and so learning aural identification is a key musical skill that participants should develop, and which would facilitate future musical efforts, whether learning musical notation or improvisation, or enhancing music appreciation, such as following structural elements.

The types of music-making a learner engages may also affect their rate of musical progress. For example, school-age students have been demonstrated to have better performance skills when improvisation is included [23]. Improvisation can also form part of tests to assess overall musical performance skills [24]. Since improvisation relies on auditory memory for storage and retrieval of musical patterns, we wanted to understand how the inclusion of improvisation training might affect older adult learning. Improvisation may also permit earlier individualisation of music-making than replication of compositions, because of its flexible yet potentially systematic approaches [25,26], applicable in many spheres.

This paper details quantitative measurement of musical learning by older adult novices across a 12-month period, and its subsequent retention over a further 6 months. We assess participants’ learning of aural perception skills and memory for pitch and melody, as well as their use of these skills for performance of pre-composed music and for improvisation.

## Participants, materials and methods

This study received Human Research Ethics approval from Western Sydney University (H13206).

### Participants and training program

Sixty-eight participants were recruited subject to the following inclusion criteria: i) aged 65–80, ii) no more than 2 years previous formal musical training and no more than 2 years playing experience on any instrument. We did not exclude some greater experience in singing performance, but the Supplementary Material confirms our participants were musical novices, and low in singing expertise (data from Goldsmiths Musical Sophistication Index questionnaires, as below); and iii) cognitively healthy as measured by the Mini-Addenbrooke’s Cognitive Examination (M-ACE, score > 25) [27]. Sixty-four participants were right-handed, two left-handed, and two ambidextrous. Lessons for the first participants commenced in November 2019, while an additional nine groups of participants followed by rolling recruitment. The final group concluded lessons in June 2022. Across the 12-month learning period, all participants received six months training on each of two different instruments: a digital piano keyboard (Yamaha PSR-E363), and the iPad touchscreen app, *ThumbJam* (https://thumbjam.com), with instrument order between groups randomised and counterbalanced. No keyboard pedal was provided, and there was no training in pedalling. Tasks included both reproducing pre-composed melodies (containing familiar and unfamiliar material), as well as improvising (i.e., creating new material). In both cases performance and training was limited to single-handed melodies, played with the right hand only. Throughout the 12 months study, there was a 3 monthly alternation of improvisation and replication learning, with the starting condition also randomised. To facilitate home practice participants were loaned both instruments for the entire 18-month period. All instruction used aural prompts rather than any notation, and emphasised pitch/pitch sequence rather than rhythmic precision.

Apart from a very small number of sessions up to March 2020 [19], all teaching was online in groups using *Zoom* (https://zoom.us). A dedicated technical assistant provided individual support concerning Zoom and instrument set up and MIDI-recording, joining all scheduled lessons and test performance sessions to support the teacher, and available between times for one-on-one support for any hardware/software technical issues. The assistant was also amongst the researchers who participated in the Test (performance) session break-out rooms with individual participants performing (see Data collection, below). An arrangement was made to record all of the online lessons and make them available to participants (time-limited and password protected) so these could be used in the event of difficulties with connection, although this was rare. The net result was a very high effective attendance rate while enrolled in the classes. The response to the online group approach, and the roles of our technical support assistant are discussed in more detail in (MacRitchie et al., 2023).

#### Participant summary.

AMME comprised 68 cognitively intact older adults (aged 65–79, *M* = 70.3, *SD* = 3.8 years; 60 female, 8 male). We attempted specific approaches to recruit more males, but with very limited success. 70.6% of participants reported having no prior musical learning experience. Participant drop-out was relatively low (13/68, 19.1%). The ten groups each contained between 6 and 8 members at onset, group size *M* = 6.8. Further participant details are in the Supplementary Material.

### Data collection procedure

Data on participants’ achievements and views were recorded through i) musical performances self-recorded during lessons and after each teaching block, as MIDI files, ii) a battery of cognitive and motor tests, occurring every three months from baseline and over the 12 months teaching and 6 months thereafter, and iii) audio recordings of semi-structured largely qualitative interviews about their experience in AMME, which shortly followed each cognitive test battery. The cognitive tests (forthcoming) included the Digit Span Test, Alternative Uses Task, and Trail Making Test B, together with a group of questionnaires. The main motor tasks were single and dual Finger Tapping Tests, and Trail Making Test A. The last cognitive/motor tests for the final group occurred in December 2022, six months following the final lessons. The interviews (point iii) aimed to elucidate engagement with the music lessons, group dynamics, and practice strategies (structure details are available in Supplementary Material to MacRitchie et al., 2023). These interviews were conducted by authors JM and RTD, who took no part in the cognitive and motor measures (to minimise experimenter bias). All cognitive and motor tests were done by author AC, who had no knowledge of group order as described below. Data collected in ii) and iii) will be fully analysed in forthcoming papers. Data used here will be made available on OSF immediately upon publication of this paper, together with guidance and other related information.

### Training and testing flow

**Fig 1:**
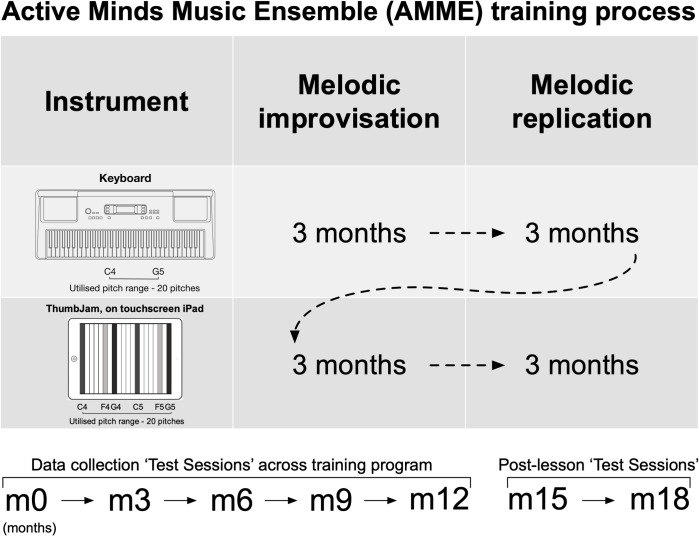
Overview of the AMME teaching program.

Fig 1 summarises the training and testing flow. The main data collection occurred at ‘Test (Performance) Sessions’ (one or more interactions with the experimenters), with the first being at baseline (prior to the first lesson: ‘Test Session m0’, i.e., month zero), others at the end of every 3 month block. Therefore, Test Sessions m0 and m12 encompass the teaching, Test Sessions m15 and m18 the period after the teaching. Testing Sessions m3–m12 included one-on-one ‘Session Performances m3–m12’. The counterbalanced cross-over (within participants) design was such that for each participant group, every block or post-session 3-month period had a unique description in terms of time segment number (4 units across training, 2 thereafter), keyboard or iPad use, replication or improvisation training counts. Given the counterbalancing of group orders (see Fig 1) this also ensured there was no overall co-linearity between any of these significant performance predictors, and models of the data could be unambiguous. The 5 items performed in each Test Session Performance (detailed immediately after Fig 1) are termed ‘Performance Items’ 1 to 5. The Session Performances were intended both as data gathering and to impart continuing group enthusiasm (they were framed as a ‘celebration’). As mentioned already, standardised qualitative interviews (delivered by JM or RTD), and cognitive tests (delivered by AC) occurred shortly after Test Sessions m3-m12, usually in direct succession to each other. The cognitive tests were also delivered at m0.

Each of the four ‘3 month’ quadrants represents a teaching block that focused on a particular instrument and task, with the full four blocks equating to the year of learning (with the specific ordering of instrument and task randomised and counterbalanced across the ten groups). The diagram illustrates one possible sequence, given that for the counterbalancing, the Instruments were adopted for 6-month blocks, while the musical approaches were adopted for 3-month blocks. During each 3 month block, 6 one hour lessons were given, and practise foci prescribed for the upcoming period. The ‘Test Sessions’ comprised the Performance session (with 5 successive performance items as described in the immediately following text), and the test battery and qualitative interviews (normally done in immediate online apposition to each other, but by AC (test battery), and either JM or RTD (interviews)).

As just noted, between blocks, in a ‘Test (Performance) Session’ participants played 5 successive ‘Session Performances’, each supported by an individual researcher online (including our technical assistant, and occasionally two others) in a Zoom break-out room. For each of the 5 Performance items, participants were given 2 minutes practice time before a MIDI recording was taken, with the following procedure:

1. A favourite rehearsed song (or in the case of improvisation blocks, they could render an improvisation they had been developing).
2. Returning temporarily to the group meeting room, the participants communally chose a tune from a possible bank that they thought they remembered hearing (outside of lessons), but had not studied. Then in individual break-out rooms they attempted to play this melody.
3. They were given 3 brief aural exposures (individually, in the break out rooms) to a recording of the unstudied melody while experimenting with their keyboard at rendering it during the 2 minutes practice time, and then made a further attempt at performing it.
4. Remaining in the break out room with their researcher-supporter, they performed this and the following item. For item 4, they improvised freely on their own choice of a melody fragment. We suggested participants chose a simple short sequence of 3–4 notes, from or related to item 3.
5. They improvised on the fragment, but specifying selected improvisation tools out of the list of 14 (we recommended using 1–2 tools at most, see below for details).

For cases where a participant undertook improvisation, Session Performance items prior to receiving any improvisation training (i.e. if they had only received replication training thus far), they were given brief explanations of the improvisation tools listed above. Informal recordings of classes were also made in lessons 1, 2, 4 and 6 of each training block (to ensure participants were familiar with the recording process by the time of the Test Performance Sessions). These data are not included in present analyses. All recordings were made as MIDI files, so that these could be readily extracted as symbolic numerical files for analysis.

The training focused on aural skills and their translation into simple performances, by attention to the two key musical functions of replicating material and generating it (improvisation) on two separate hand-driven instruments. A Yamaha electronic keyboard presented a conventional velocity-sensitive piano interface and sound. As shown in Fig 1, the second listed instrument, ThumbJam, was set up to present a single row of 20 chromatic keys on the screen, with no discrimination between black and white notes, but displaying three coloured bands within every chromatic octave, indicating the pitches of C, F, and G. Each key occupies the whole height of the landscape-oriented screen apart from some menu items, and the sound becomes louder as the touch position moves towards the top of the screen. Everything was monodic (single strand), and realised on sampled piano sounds. The two keyboards require overlapping, but different motor skills.

For replication learning a set of pre-recorded well-known simple pieces (see Supplementary Material) were taught, together with a small group of specially composed pieces that extended the melodic, implied harmonic, and rhythmic diversity of the well-known pieces. Task instruction focused on replicating melodies with particular emphasis on aurally identifying pitches and their sequence. Commonly, fragments of three to five notes were learned separately and then joined together to form the single right-hand melodies (maximum 30 notes). After a certain level of sequence precision with a melody was achieved, our teacher would indicate that performance might be enhanced through ‘expressive timing variation’; through the use of dynamics (changes in performed loudness); and/or through articulation (‘changing the acoustic connectedness of notes by means of the length of sonic gap between them’). No attempt was made to explain music theoretic components such as meter or tonality, unless a question was raised, when it was treated simply and briefly.

A set of 14 improvisation tasks were taught [25], where in each task a melodic fragment is subject to limited systematic variations that may partially overlap. Reference was made to pitch contours. As for the replication learning we sought self-chosen improvisation outcomes without reference to tonality or metricality, in single (right) handed melodies (C4-G5). Participants were given an aural prompt of three to five notes, and asked to improvise on it for a maximum of 30 notes. The methods, presented on-screen during teaching and Session Performances, were:

1. Adding repeated notes
2. Adding passing notes (in between) or neighbour notes
3. Change the distance between the notes
4. Change the note lengths (Rhythm)
5. Make it louder or softer (Crescendo or Diminuendo) (this was explained as gradual change)
6. Make it suddenly change volume (Accents) (this was explained as abrupt change)
7. Put silences in between notes (Rests)
8. Try playing at different speeds
9. Play smooth and joined (Legato)
10. Play bouncy and short (Staccato)
11. Different combinations of Legato and Staccato (Slurring)
12. Reverse the notes (Retrograde) (this refers to reversing the note order).
13. Repeat higher or lower (Sequence)
14. Change the set of notes/ include some black notes (Modulation)

Throughout the teaching and other interactions, technical terms were kept to a minimum (staccato, legato, and retrograde were occasionally used as shorthand for a definitional phrase), and given no in-depth elaboration (e.g., ‘modulation’, method 14). To attempt to give a flavour of these fairly simple methods for a non-musician, we elaborate here slightly on items 5 and 10. Item 5 requests a gradual increase or decrease in performed loudness. With the digital piano keyboard, this is achieved by striking the key with changing velocity, which digitally mimics the effect of a piano hammer hitting the strings with different speeds: faster keyboard depression being louder. For this to be a gradual change in loudness requires quite refined physical control to be developed. With the iPad, a different system is required, where control is by the point of finger impact (from bottom to top of the landscape screen, soft to loud) on the portion of the screen corresponding to the chosen note (20 notes are available in our system, arranged L to R across the screen as shown in Fig 1). So an increase in volume is achieved by gradually moving the attack point up the screen, and vice versa for a decrease. This requires a different kind of dexterity from that on the digital piano keyboard: for example, the fingers can be turned away from being at right angles to the bottom edge of the screen, and/or they can be moved between attacks. Item 10 requires short notes. This means on both instruments that a note is attacked and sounded by touch, and then released quickly (decaying quickly to silence) rather than being held down for a while (which sustains the sound).

The repeated cognitive and motor function measures, and the qualitative interview data (see Fig 1) are not largely discussed here, but some of the included musical measures and questions were 1) a Pitch Direction Task—used to encourage people with difficulties in this respect to undertake self-driven training [28]; 2) three open-ended questions used to elicit responses on Musical Possible Selves [1], to be discussed elsewhere; and 3) three items from the Goldsmiths Musical Sophistication Index (henceforth GMSI). These items were the Self-report questionnaire [10], the Melodic Discrimination Test, MDT [29] and the Computerised Adaptive Beat Alignment Test, CA-BAT [30]. MDT measures whether a participant can distinguish two melodies presented in rapid succession, while CA-BAT considers whether a metronome pulse is aligned with accompanying music. MDT and CA-BAT were each used with default settings.

### Modelling the automated musical performance measures

We previously devised ‘AMMRI’, a computational suite of precise ‘automated measures of musical replication and improvisation’ in sequences recorded in MIDI [31]. These and other analyses were performed in R version 4.4, with particular use of the brms package for regression modelling, and marginal effects for quantitation of individual effect sizes in complex models (see Supplementary for much more detail). Core to the assessment of replication fidelity was dynamic time warping (henceforth ‘dtw’) which allows one to assess the distance between a participant performance and the original recording (‘reference’) of the material being performed. Dtw identifies the segment(s) of a reference being rendered, and measures the distance between performance and reference. Since a performance of a sequence of 5 correct notes out of a piece of 15 notes is a lesser achievement than the performance of 15 out of 15, a similarity score (1 – distance (scaled to 0–1)) is then multiplied by the proportion of the reference whose notes are matched (that is, approximated) by the performance. Similar measures can be made of note timing similarities, and are discussed. For the assessment of improvisation, code was written to assess each specified method, sometimes in more than one respect. Throughout, we place more emphasis on the assessment of pitch sequence than timing or dynamics, corresponding to the relative attention paid during the teaching.

Bayesian statistics (see Supplementary) provide full determinations of the modelled distributions of coefficients rather than simply point estimates. They allow both positive and null hypothesis testing, and the ‘evidence ratio’ in favour of a hypothesis is the ratio of the probability distribution area on its positive side to that on its negative, and a strong ratio (>19 for one sided tests) is thus a >95:5 ratio [32]. The Bayesian approach also allows developing combined measures, where several overlapping aspects of the same learning process are measured, as is common in educational research. In all cases we could obtain good models with *SD* of the prediction error around 10% of the measured value, and a Bayesian R^2^ usually >0.5 (see Supplementary).

It is useful to have abbreviations for some of the key methods, variables and predictors we present in the modelling results to follow: we therefore list them here, and they are further elaborated as appropriate when used:

brms: the R package Bayesian Regression Modelling with Stan.

ctimprep: sequential counts of 3 month blocks of improvisation or replication training, in format improvisation-replication i-r, where i and r can both range from 0–2.

dtw: dynamic time warping. Using the R package dtw, we made assessments of the similarity of replication performances to the source melody.

imprct: count of the number of blocks of improvisation training a participant has experienced.

kbipI and kbipK: indicates training blocks where the kbip (keyboard-iPad) instrument used was respectively iPad or Yamaha keyboard.

klpitchsim and klioisim (6) are versions of Kullback-Liebler distributional divergence measures (between replication and original), adjusted to range 0–1, the first for pitch, the second for timings. Since they are distributions, they do not need length adjustment.

nimptasks: the number of improvisation tasks participants chose to try to apply during Test Performance Session Item 5, where we recommended not more than 2, but many people claimed more when asked immediately after each performance.

palignedprop (from the dtw assessment) is the proportion of a target melody that could be aligned with (find a close counterpart in) the performance.

pitchdtwladj is the pitchdtwsim adjusted by multiplying by the corresponding palignedprop (so that fair replications of short sections attract lower scores than comparably fair replications of longer sections).

pitchdtwsim is the dynamic time warping (dtw) assessment of the performed pitch sequence’s similarity to that of the target piece (range 0–1).

pioidtwsim, pioialignedprop and pioidtwladj are the dtw measures that jointly consider both pitch and note timings (ioi = inter-onset interval, the time between successive events), and correspond to pdtwsim, palignedprop, and pitchdtwladj.

repct: count of the number of blocks of replication training a participant has experienced.

## Results

The MDT and CA-BAT, measured 7 times at 3-monthly intervals were our most systematic assessment of musical learning, focussed like the teaching on musical aural skills. These results are presented first.

### Formal tests: Aural skill learning

**Fig 2:**
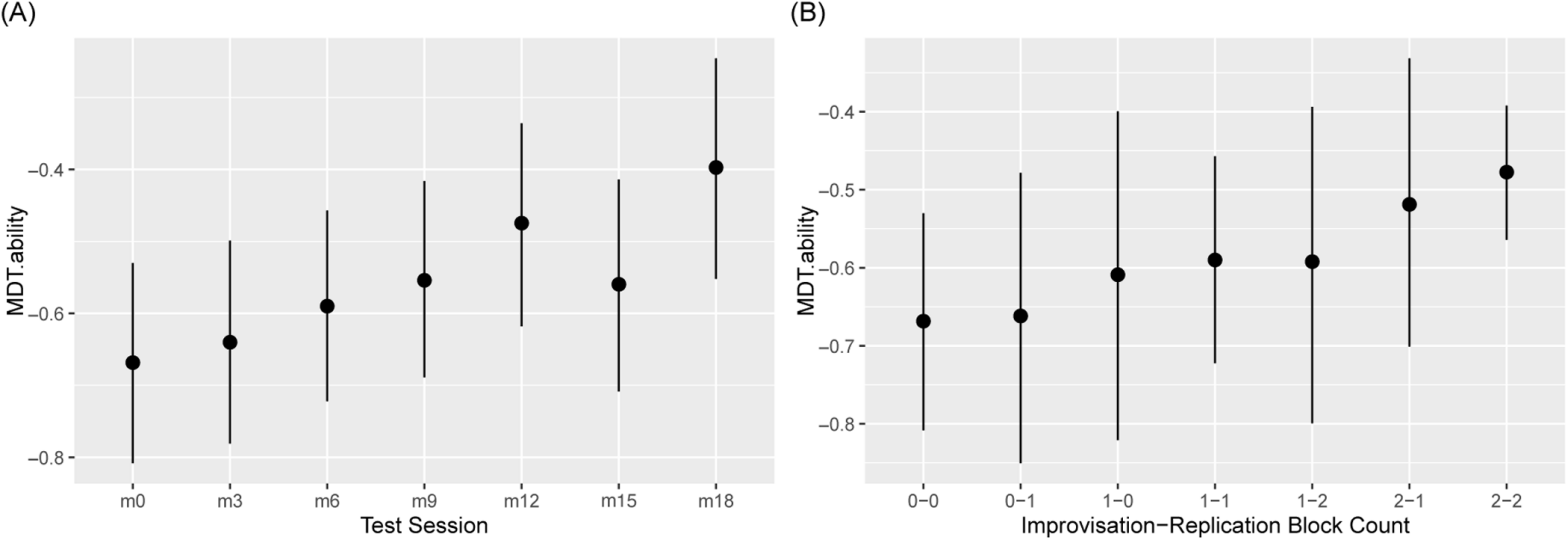
Melodic detection across the AMME study. (A) MDT according to Test Session: In the left-hand descriptive graph, m0 (Test Session at month zero) was the pre-test, preceding the four blocks of learning (m3-m12). Test Sessions m15/m18 followed the cessation of teaching at 3-month intervals. Bayesian Credibility Intervals (BCI) and their medians are shown, as in later figures. (B) MDT ability by Improvisation and Replication block counts: In the right-hand analytic graph, the Improvisation-Replication counts are ctimprep, format i-r. The substantial improvement by Test Session is supported by strong evidence ratios (257, 35) for m18/m12 vs m0. Other comparisons with m0 were weak. The inconsistent differences amongst Test Sessions m12-m18 were weakly evidenced (ratios <15.8). In the Improvisation-Replication graph, point 2-2 was greater than 0-0/0-1 (strong evidence ratios 92.0 and 23.8 respectively).

We hypothesised that both the replication and improvisation blocks would contribute to aural skill development, and hence formed a model of our data with the following key predictors tested: ‘Test Session’ (from m0 – m18 in the formal tests), and ‘ctimprep’, the sequential counts of improvisation and replication training (format imp-rep, both values 0–2). The mixed effects multilevel model formulae for the measures, optimised as far as possible for interactions and group effects, are detailed in the Supplementary Material. Melody detection was enhanced during learning (Fig 2).

Critical assessment of Fig 2 confirms improvement in melodic discrimination during learning, and retention for 6 months after. The learning seems progressive, but this should be viewed cautiously given weak evidence ratios in several cases. But if Test Session is treated as a continuous numerical (rather than factor) variable (1–5) for the learning period, a strongly-evidenced positive coefficient can be observed. Consistent with this, the drop in performance between Test Sessions m12/m15 is not strongly evidenced, and is best interpreted as simple variability. In additional models, m0 MDT values (i.e. pre-learning ability) were strongly evidenced positive predictors of subsequent outcomes, but did not improve model quality, when account was taken of the consequent reduction in dataset size. Overall, information was lost by using these baseline values as a predictor: this approach was not pursued.

Fig 2 RH shows only a small increase in MDT ability after the first replication block, and additional increase after the second block. There is a larger increase after each improvisation block. These *marginal effects* predictions set all the variables other than that specified on the x-axis to their relevant average or modal values. This means that these other variable values also change by Test Session. While the effect of the addition of a single block of training does not change the reference value for the other training mode, it does change the Test Session count (i.e., the amount of social interaction and time passage). It seems clear from Fig 2 that virtually all change by Test Session is explicable by the training, with Improvisation being the larger contributor. That test sessions (and therefore time of social exposure to the online groups) per se cause minimal change is supported by considering joint changes by Test Session and ctimprep (not shown): after the first test session, one of the two training approaches was incremented, and one stayed constant, hence one can again observe that the numerical difference between MDT for session n and n+1 for the unchanging training approach was much lower than that for an increment in replication, and poorly evidenced.

**Fig 3:**
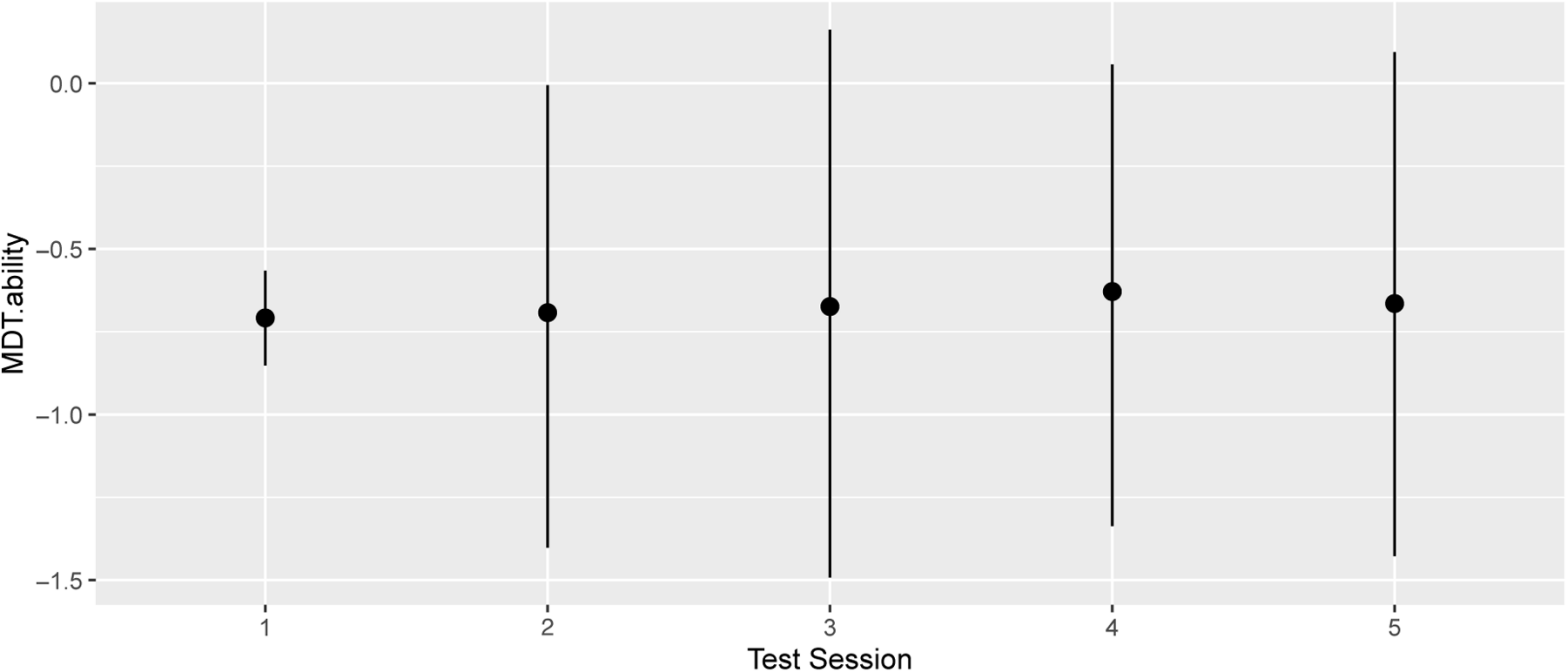
MDT.ability by Test Session count alone (counterfactual).

To assess this further, we modelled successive sessions counterfactually, with no change in either training count, by prediction from the Bayesian Posterior: Fig 3 confirms that Test Session count during m0-m12 (i.e. time passage plus social exposure, but with no training) does not affect the score.

For these counterfactual predictions, ctimprep is static at 0–0 (no training), and all participants are included. The latter four conditions described are counterfactual (did not occur), but can be predicted because they fall within the ranges of the data represented in the model posterior.

The range of median estimates in Fig 3 is -0.708 to -0.629, much narrower than in Fig 2 (-0.668 to -0.477 for changing ctimprep), confirming that the predicted effect of increasing Session per se is minimal. Since none of the values were strongly evidenced as different from others (most hardly evidenced at all) we conclude that Test Session per se is not an effector of MDT learning. This conclusion holds later in each of the sequential analyses we present, and is not detailed further. This prompts a further question: does improvisation have more positive effect than replication, as implied by Fig 2? Using the model posterior, the total MDT.ability increment associated with the improvisation sessions was 0.19, that with replication was 0.06, the measured difference 0.13 (with a weak evidence ratio, 2.83 against a possible zero value). Thus, the data indicate a greater impact of improvisation than replication, but they are not statistically conclusive. Further counterfactual analyses of the impacts of improvisation and replication without Test Session change supported this interpretation but remained weakly evidenced.

Next, CA-BAT (Beat Alignment) was examined with a similar approach and a good model was obtained (not shown). Using the *marginal effects* library to extract the CA-BAT scores from the posterior with respect to Test Session showed erratic performance, and no Test Session value higher than that of the baseline, Test Session m0, which was -0.743 (95% BCI -0.922/-0.572). Indeed, two Test Sessions were strongly evidenced as lower. Correspondingly there were no strongly evidenced changes in score with respect to improvisation and replication training. We conclude that beat recognition was not systematically changed during our training, consistent with the teaching emphasis on pitch aspects, and also with results to follow.

### Test session performances: Replication and improvisation performance learning

#### Replication Learning.

Eight assessments of replication accuracy and aptness (comparing performance MIDI data with that of the attempted piece [31]) were made. For the present purposes, each measure was scaled to a range 0–1, so they could be fairly weighted against each other. ‘pitchdtwsim’ (1), is the dynamic time warping (dtw) assessment of the performed pitch sequence’s similarity to the target piece (ranging from 0, totally dissimilar, to 1, identical). Dtw does not penalise for the proportion of the tune actually performed so we measured that proportion, the ‘palignedprop’ (2) (again from the dtw), and made a ‘length adjusted’ dtw measure ‘pitchdtwladj’ (7), by multiplying pitchdtwsim*palignedprop, so reducing the similarity score with lower extents of replication. ‘pioidtwsim’ (3), ‘pioialignedprop’ (4) and ‘pioidtwladj’ (8) are the corresponding dtw measures that jointly consider both pitch and note timings (ioi = inter-onset interval, the time between successive events). ‘klpitchsim’ (5) and ‘klioisim’ (6) are similarity versions of Kullback-Liebler distributional divergence measures, adjusted to range 0–1, the first for pitch, the second for timings. This distributional measure does not concern sequence accuracy but similarity of the pitch set used: a basic measure of stylistic similarity between performance and target.

Given emphasis on teaching pitch sequence we considered pitchdtwladj the most critical indicator. A few points can be made directly from its Model (#2) estimates (see Supplementary). Here Test Session Performance Items 2 (coefficient estimate -0.37) and 3 (-0.24) have lower scores than the dummy variable Performance Item 1 (strong stated Evidence Ratios (ER) of infinity, 1332.3 respectively). The ER for a negative difference between the coefficients of Performance Items 3 vs 2 was also strong (234.3). Thus as expected, Performance Item 1 was rendered most accurately of the three (being pre-rehearsed), while the poor score of Performance item 2 was somewhat improved by relistening and practising Item 3 over a short period followed by a repeat performance. This provides clear indication both of medium- (i.e. during the preparatory period) and short-term (i.e. during the Performance Session itself) learning by listening and aural preparation during practice. Fig 5 (later below) details this. There was also strong evidence that the coefficient on keyboard sessions (kbipK) was higher than that for the dummy variable iPad sessions (kbipI: ER 28.09). This coincided with a greater reported liking for the keyboard than the iPad.

**Fig 4:**
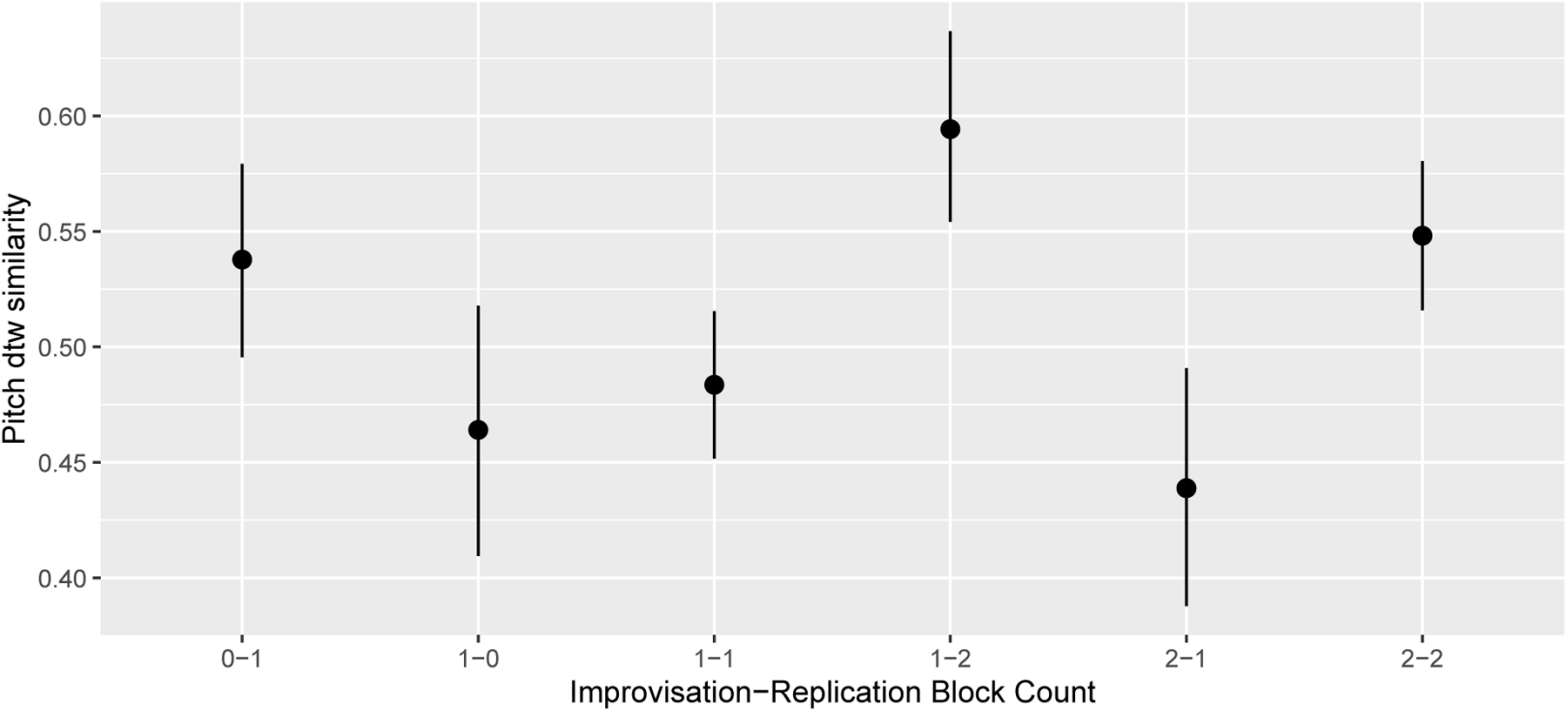
Replication pitch similarity score by Improvisation and Replication training block counts. Note that in the improvisation teaching blocks, the pieces for Performance Item 1 were normally a pre-prepared improvisation, and hence are not included in the data here (see below for their consideration in relation to improvisation).

Fig 4 shows the dependence of pitch replication precision on the specific training blocks (ctimprep).

In Fig 4 the comparison of replication count *n* with *n*+1 (with unchanging improvisation count) shows an increase in median value. Two of these have strong evidence ratios which are 2.65, infinite, infinite, infinite for, respectively 1–1 vs 1–0,1–2 vs 1–1, 1–2 vs 1–0, 2–2 vs 2–1. Conversely, the comparisons for an increase in the improvisation count show numerical decreases, again in two cases with strong evidence (sequentially ERs of 49.63, 14.44, 21.47). The improvement in the replication blocks is consistent with the training, but the decrement due to improvisation is surprising, perhaps indicative of simple decay, or perhaps of a negative impact of the freedom of pitch choice encouraged by improvisation (given the recurrent evidence of insubstantial effects of Test Session count, i.e. time/social interaction per se).

**Fig 5:**
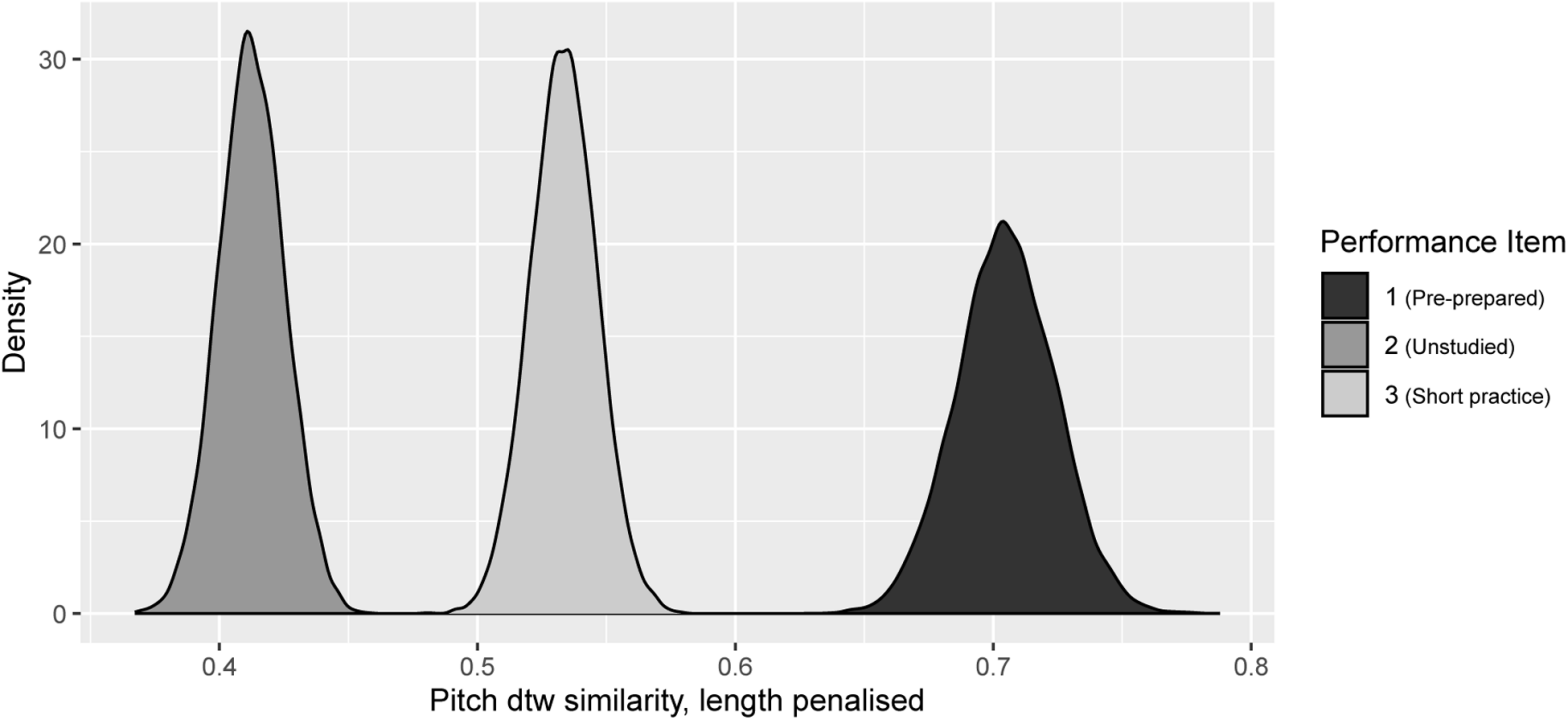
Pitch score (pitchdtwladj) according to Performance item. As described in Methods, Performance Item 1 was a pre-prepared rendering of a studied melody, 2 was a first time rendering of a newly communally chosen melody (unstudied), and 3 was the result of practising and re-listening to melody 2 for about 2 minutes. Evidence ratios for the differences were reported as infinite in each case.

Fig 5 uses the model posterior distribution to confirm that performance 1 (pre-prepared) was best, and 3 (after short practice), was better than 2 (no practice).

Thus Figs 4/5 together show that there was long-term learning of replication skills, and the best performances occurred after 2 blocks of replication training, exceeding that before training, while there were apparent regressions between during the improvisation blocks. There was also short-term learning of newly studied melodies. Separate analyses again showed no effect of Test Session count per se.

The Kullback-Liebler distributional pitch similarity measures a different kind of aural sensitivity from that of measures 1,7. With a slightly weaker Bayesian model (Bayesian R^2^= 0.45) from the same predictors as above it showed qualitatively identical patterns to those described; though with a narrower and higher score range (all >0.9). Strong evidence ratios characterised the same contrasts as just discussed, with very minor exceptions. This suggests there was learning of generic pitch structure, as well as specific sequences. Because very short performances would give poor distributional information, we did not length penalise this parameter.

The third measure potentially indicating replication learning is the combined pitchioidtw measure. The measure was successfully modelled by our standard broad model (sigma 0.06, Bayesian R^2^ = 0.45) and the Performance Item effects described already occurred with very strong evidence ratios, while the pattern of impact of the replication and improvisation counts remained also, but damped, and with weaker evidence ratios. The klioisim measures, detecting the timing distributional similarities alone, showed no change with session item or replication and improvisation counts (nor, as usual, of session per se); effects of instrument were very small. We conclude from consideration of both pitchiodtw and klioisim that inter-onset timings were only modestly learned if at all, in agreement with the earlier formal CA-BAT. As mentioned, a combined pitch sequence and distributional similarity measure showed higher scores but similar patterns to Fig 4 (see Supplementary). Given the model, this combined measure can be predicted for an individual at any chosen point in the experiment, and hence used as a predictor in cognitive and motor models in future work, as a direct indicator of the possible impact of learning per se, as could the MDT measure.

### Learning to improvise

**Fig 6:**
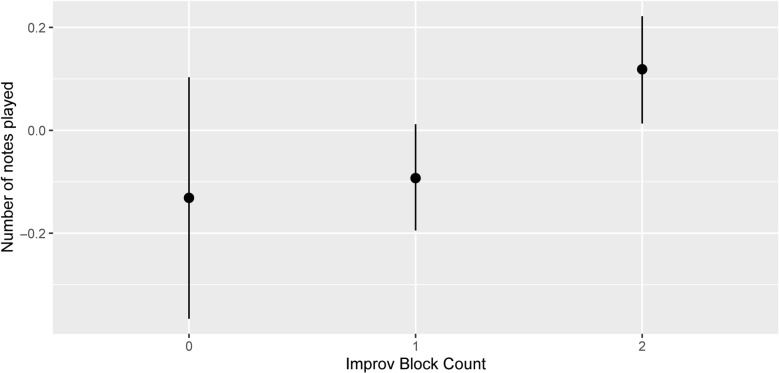
Number of improvised notes by improvisation count. Posterior sampling of a model of note extent during improvisations. Improvisation Block Count 2 was strongly evidenced as greater than counts 1 or 0 (evidence ratios 499, 35.9 respectively), while the other comparison was only weakly evidenced.

We anticipated that the extent (number of notes) of performed improvisations would increase during the improvisation training, likely reflecting some combination of enhanced capacity, confidence and enthusiasm. Fig 6 confirms this using posterior sampling of a simple model of that parameter (sigma 0.75, Bayesian R^2^ = 0.45) from the data concerning Performance Items 4 and 5: the improvisation data were standardised (*M* = 0, *SD* = 1) because it was less suitable to compress them all into the 0–1 range as was done for the replication results. Notes played also increased strongly in Session Performance m9 and m12 as compared with m3 and m6 (not shown), as expected. There was also strong evidence that Performance Item 5 (with tasks) attracted more notes per performance than Performance item 4: the tasks thus seemed to be an encouragement, as intended.

The measures (‘scoretypes’) we applied to the improvisation task analyses were as follows [31], where Mn indicates that the measure had selective (but not necessarily sole) relevance to improvisation method *n* as shown in Methods.

1 ‘NumNotes’: how many notes the improvisation contained
2 ‘M1pintervaldivers’: diversity of pitch intervals used
3 ‘M1pintervalrange’: range of pitch intervals used
4 ‘M2ioidivers: diversity of note inter-onset intervals (ioi)
5 ‘M2iorange’: range of note inter-onset intervals
6 ‘M3pitchdivers’: diversity of individual pitches used
7 ‘M3prange: range of individual pitches used
8 ’M4repeatnotes’: the number of occurrences of immediately repeated notes
9 ‘M5Passnotesprop’: the number of occurrences of passing notes (notes that are close to the antecedent note)
10 ‘M6crescdim’: the count of crescendos and diminuendos
11 ‘M7AccentProp’: the count of accented notes as proportion of total
12 ‘M8silenceprop’: the proportion of performed time that was silent
13 ‘M9windioirange’: measuring average iois across a group of notes, and then analysing the range of such averages
14 ‘M12VaryStaccLeg’: the extent of staccato and legato variation
15 ‘M13retronotes’: the number of notes involved in melodic retrogrades
16 ‘M14sequences’: the number of notes involved in sequential repetitions
17 ‘M12aStaccLegabsdiff’: the average absolute duration differences between adjacent notes as proportion of the relevant ioi. An absolute value is taken to ensure that each measure and the overall result is positive
18 ‘M9aWindioiabsdiff’: the mean of the absolute differences between successive windowed estimates of average ioi.

**Fig 7:**
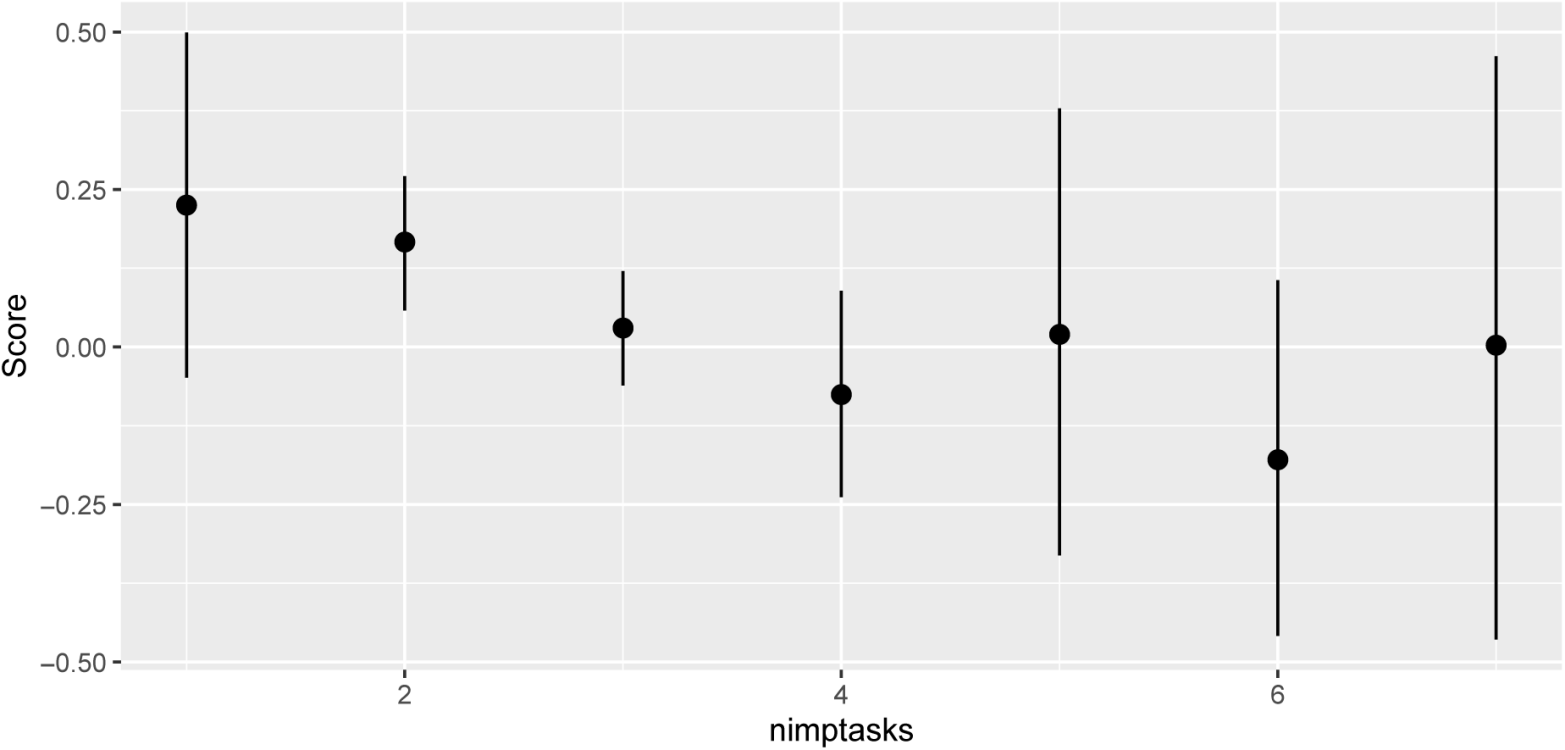
Scores in relation to the number of improvisation tasks (nimptasks) claimed. All renderings of Performance Item 5 were taken, all tasks were claimed by at least a few participants, but only the most pertinent 11 scoretypes (see text) were used. The model had sigma 0.63 and a Bayesian R^2^ of 0.62. The task: scoretype assignments were: 1 8; 2 9; 3 3; 4 4; 5 10; 6 11; 7 12; 8 18; 9 17; 10 17; 11 17; 12 15; 13 16; 14 3 (some tasks share a scoretype).

Analysis of the improvisation data in which participants specified their tasks (Performance Session item 5) was hampered by two facts. First, that in spite of our recommendation to attempt 1–2 methods at once, many reported (‘nimptasks’) using more, yet unsurprisingly could not fulfil many at once; and second that if we selected only observations with low nimptasks, and used only the pertinent measures, the sample size usually became small and unevenly distributed across blocks while still involving many different tasks. For example, in a model of four key pitch measures taken together, the best improvisation score was when nimptasks = 1, and most other values (up to nimptasks = 7) were strongly evidenced as lower. This confirmed that participants could not successfully undertake many methods simultaneously. In that model, the improvisation blocks showed a mean score of 0.151, compared with -0.04 for the replication blocks (evidence ratio 65.67), indicating a positive effect of the improvisation training. Fig 7 shows another comparison of the effects of nimptasks, using only the 11 measures selective for the complete set of 14 tasks involved: a combined ‘Scores’ measure was made from the 11 scoretype values using the variance sharing approach described in the Supplementary Material. Other median scores are lower than those for 1 or 2 tasks, with strong evidence ratios for 1 vs 4 (28.2) and 1 vs 6 (35.6) nimptasks.

**Fig 8:**
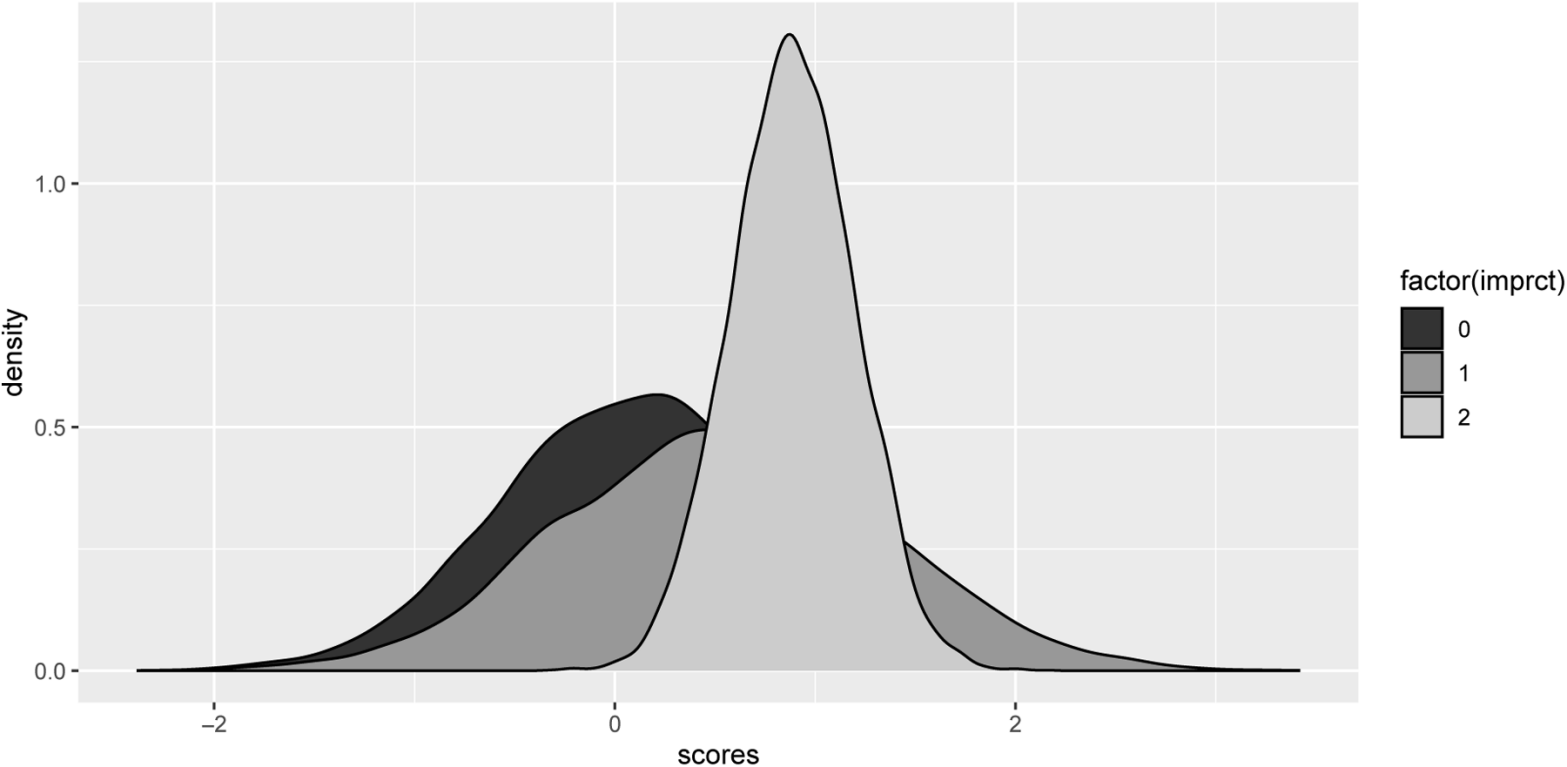
Session item 5 pitch use scores by improvisation block count (imprct), when there was 1 Improvisation task, and replication block count was fixed at 2. The model (for nimptasks *=* 1) is defined in the Supplementary Material. The evidence ratio that imprct *=*2> imprct*=* 0 was 4.97 (weak). When nimptasks 1:7 or subsets thereof were included in the data, positive differences remained with imprct 2, 1 > 0 and usually 2 > 1, but in all cases with lower evidence ratios. Note that some of the conditions interpolate from those applied: for the prediction shown we set keyboard, learning block 4, replication count 2, and the number of improvisation tasks chosen 1.

Continuing with Performance item 5, given there was no 0–0 improvisation count (imprct): replication count condition, the key choices were to compare nimptasks=1 with nimptasks 1:7 together, and to compare replication count 0 and 2, as the background on which imprct of either 0, 1 or 2 are superimposed in a counterfactual prediction. Fig 8 shows the mildly positive result obtained with repct = 2.

These cautious Bayesian analyses, though restricted by our data, provided evidence that improvisation methods were acquired, particularly on the keyboard. In addition, with Performance Item 5 under the conditions of the analyses of Fig 8, and still focussed solely on one improvisation task, predictions for improvisation tasks 9 and 14 both gave score increasing with imprct (moderate evidence ratios of 14.78 and 10.19 respectively). We next address the performance of some individual improvisation tasks further.

### Assessing whether participants learnt the individual improvisation tasks

Consequent on the limitations mentioned already, the number of participants undertaking each task within Performance Item 5 is necessarily limited (from a maximum of 51 for task 1, to 2 for task 11 and 5 for task 6). Thus our Bayesian approach was to isolate in turn the data for each task (necessarily within Performance Item 5). Then we selected only the data rows that applied the single most pertinent improvisation measure amongst our 18 measures (see Fig 7 caption). As noted already, measures 13, 14 were eliminated from consideration, and 17, 18 were preferred instead. We chose to model each individual task data set using a standardised approach, taking due account of the fact that for each model there was only one scoretype as well as solely one focus task:

score ~ kbip + imprct*trainingblockcount + repct* trainingblockcount + nimptasks + group + (1 | pid).

The resultant model posterior is used for counterfactual predictions, with nimptasks set to the ideal 1. For predicting the effect of imprct, repct is here set to 0, and vice versa (instead of the standard *marginal effects* approach). Supplementary Table 3 summarises the results. It shows weak indications of learning in improvisation blocks, usually in the first such block, for Tasks 1–6, 8, 12 and 13, of which 3 and 13 actually each show one strong evidence ratio. Positive signs for the replication blocks occur only with 1,3, 5–8 and 12 (all being cases shared with the improvisation positives, and with generally smaller effects). Task 11 could not be assessed, but overall it seems that all but 3 of the remaining 13 were learned to some extent. The somewhat erratic distribution of the positive effects of improvisation and to a lesser degree replication across the training blocks is not surprising, given the sparsity of data: this means that focus on a particular task is rarely followed through by an individual participant across sessions, rather a task is only attempted in one short learning phase. Overall, as noted we chose to give flexibility of task to encourage enthusiasm for learning, and so sacrificed some control of experimental design.

The Supplementary Material provides some further descriptive frequentist statistics on the improvisation learning with respect to the fourteen specified tasks and is largely in agreement with Supplementary Table 1: again it suggested a majority of tasks could be learned. A lack of evidence for speed changes coheres with our lack of teaching emphasis on rhythm; some other results (e.g. task 14) may reflect motor difficulties (and that on the iPad with ThumbJam, there is no delineation of ‘black’ notes of a conventional keyboard from white).

The Supplementary Material also shows participant self-appraised iPad and Piano keyboard usage efficiency, suggesting that both improved, yet there was an initial confidence in the appraisal of the iPad that was not shared with the less familiar digital Piano. The fact of enhancement of both measures supports our suggestion of motor difficulties and their improvement during learning; and a subsequent analysis of general cognitive and motor measures (currently in preparation) will demonstrate clear improvements in both.

Overall, we conclude that there was indication that participants could learn many of the improvisation tasks. With further data—and possibly further training—one can reasonably expect that strong evidence for success with most of the tasks could be obtained.

## Discussion

We discuss first participants’ successful musical learning and our analysis thereof, followed by an outline of how the analyses will be useful in preparation for our forthcoming models of cognitive and motor learning. We then briefly discuss developing self-expression and creativity, and other gaps in studies of learning music. Lastly, we switch emphasis from learning to teaching, and the potential utility of our measures in that context, including self-teaching.

Our participants successfully learnt aspects of aural recognition, replication of pitch sequences, and improvisation. They also learnt to produce pitch distributions related to those of the material being replicated. This success with pitch was in accord with our informed choice of teaching emphasis. The project’s music teacher agreed with our prior assessment (and experience) that rhythm is less emphasised than pitch in early pedagogy. We surveyed a selection of books and articles on music pedagogy that confirmed this; extreme examples are the traditions of teaching jazz improvisation by learning ‘patterns’ in specified scales and modes, as sequences of regular duration notes such as quavers in a 4/4 bar [33]. There is relatively little emphasis on the generation of rhythmic diversity of the melodic improviser. This complemented our prior experience of musicological publications, which are generally also more focussed on pitch than rhythm. For example, Cogan and Escot’s (1976) book seems pioneering amongst ‘books for students’ (p.xiii) in its even-handed treatment of pitch, rhythm and timbre: rhythm and time structure occupies 21% of the pages [34]. Yet musicians have emphasised analogies between pitch and rhythm in Western music at least since Henry Cowell’s 1927 volume [35], and it was illustrated in Stockhausen’s famous lecture demonstration of an accelerating rhythmic tone becoming a pitch. In some other cultures the relationships have probably been considered much longer. This underlines that our emphasis on aural appreciation and production of pitch was in some ways a necessary pragmatic choice given participant expectations, and the limited teaching time available. More balance would undoubtedly be desirable in a longer-term learning approach.

Thus, pitch learning was stronger than rhythmic learning, though some rhythmic methods were partially achieved (see Supplementary). Both short and long-term learning of musical materials occurred, with short referring to a span of minutes during Session Performances: the performance of an unprepared but aurally familiar piece improved within several minutes, after only a few listenings and some keyboard experimentation. We consider the learning achieved within one block (approximately 12 weeks containing 6 fortnightly hour-long group sessions) to be medium term: this was demonstrated by the higher performance scores for a tune that had been pre-prepared for the Session Performances, in comparison with the two Performance Items just discussed. Long term learning was evidenced by improvement across the 12 months teaching, and the preservation of abilities for 6 months.

Our cross-over within-participants design was intended to allow distinguishing effects of the different teaching styles and instruments, from effects of time in an online social context. Performance was better on the keyboard than iPad, even though participants’ claimed fluency on the iPad was only slightly increased during teaching, while that on keyboard was more enhanced. The data permit an assessment of whether pitch handling was improved in replications even by the study of improvisation alone, and vice versa. Our prediction was that improvisation training might enhance replication, while vice versa would be less apparent: the results support those expectations, though the differences were small. Professional and academically trained improvisers often note a feeling of freedom in performing that they contrast with the admitted pressured tension of classical performers who do not improvise. We speculate that perhaps our adult learners were beginning to develop a small component of such relaxation, revealed in their enhanced replication and extended improvisations, though there are multiple possible explanations that deserve consideration. Even slighter was the hypothetical effect of increasing learning block exposure without offering training, i.e. the possible component of learning that was independent of training per se: clearly such a condition did not exist in our experiment, but it could be counterfactually modelled from the posterior of an appropriate model that artificially sets the replication and improvisation learning session counts at zero while varying learning block count. Our design was successful in allowing attribution of effects of replication and improvisation training as well as showing the lack of effect of time per se. The effects of improvisation support prior claims that it can benefit musical performance and appreciation.

As noted in the caption of Fig 1, we also measured cognitive and motor outcomes (extensive publication currently in preparation). One of the purposes of our computational musical measures was to later determine whether any changes in cognition and motor function could be directly attributed to the achieved musical learning, as opposed to duration/extent of training per se. Evidence to date (currently in preparation) strongly indicates a positive answer.

It seems that musical performance can become self-expressive. One might think this would even unprompted be more of a self-aware anticipated and appealing target for older than for very young learners. This complex idea cannot be elaborated here [12], but it necessarily involves personal creativity: generating something that is new to the person, and possibly but not necessarily new to the world. That something might be an individual way of enunciating a pre-existing phrase, or if encouraged by improvisation, a way of transforming pitch and rhythm to form new structure. A few participants in interacting with the teacher described enjoyment in ways that could relate both to expressiveness and creativity, which will be part of a forthcoming analysis of our interview material. On the other hand, no-one freely used the word ‘creative’ to describe their early endeavours in music, and most were also cautious about the word ‘expressive’. Participants reported hugely enjoying the course and their efforts, so there are grounds to hope that similar training processes would enhance musical appreciation and production in the longer term. In spite of this limitation, overall our participants learned a considerable amount about both reproducing and improvising music. Analysis of our relevant data from the Musical Possible Selves questionnaire will be presented in our upcoming paper on the qualitative studies we made.

There remain many gaps in the studies of early-stage musical learning in older adults, and more surprisingly, also in studies of young learners. Our study assumed that reading notation could not be practically and reliably taught across a varied sample within our time frame, but this deserves closer empirical attention. To us, there is no doubt that learning notation is ultimately valuable, but the question is where in the most successful sequence of learning it should feature, whether for adults or young. Because of the identified barriers it presents, we believe it is important to assess scaffolding approaches to learning notation (where a simplified notation is learnt as a stepping stone to conventional notation), and then in the most successful mode of notation learning, assess comparatively where in an introductory music learning sequence it is best introduced. Related is the question of the benefits of allowing a learner to choose their own program of development in consultation with a teacher. Self-directed approaches clearly do have benefits [15], but it would be difficult to assess whether these would exceed any disadvantage that flowed from their choices.

Finally, turning from learning to teaching, we note that our suite of automated measures (AMMRI; see Dean et al. 2022) confirm that objective assessments are feasible, and indicate that they might readily be developed in an app that could easily be used by both learners and teachers. Given the increasing emphasis on music-making at home for everyone, as well as the strictures imposed by the costs of high-quality teaching, such an app could be very valuable and useful. It would hopefully not replace teaching, but rather complement it, and perhaps allow more to afford at least some personal tuition.

## Supporting information

S1 File Sample MIDI files of short melodies composed for this study. (ZIP)

S2 File Detailed description of statistical methods, and additional models and figures. (PDF)
