# Supplementary material for: Objective demonstration and quantitation of musical learning in older adult novices across a 12-month online study
Source: PLoS One. 2025 Apr 7;20(4):e0320055. doi: 10.1371/journal.pone.0320055 (PMC11975106; doi:10.1371/journal.pone.0320055)
Supplement: S2 File — (PDF) [file pone.0320055.s002.pdf]

Supplementary Material for
Objective Demonstration and Quantitation of Musical Learning in Older Adult Novices during 12 months online study

Anthony Chmiel[1](ORCID iD: 0000-0003-3294-0534), Roger T. Dean (ORCID iD: 0000-0002-8859-8902), Catherine J. Stevens (ORCID iD: 0000-0002-7558-2717), Jennifer MacRitchie[2](ORCID iD: 0000-0003-4183-6552)
The MARCS Institute for Brain, Behaviour and Development, Western Sydney University.
Current affiliations: University of Sydney, Sydney Conservatorium of Music[1]; University of Sheffield, Department of Music[2].
Corresponding author: roger.dean@westernsydney.edu.au

Supplementary information on Methods and Participants

Participant Handedness

Of our 68 participants, 64 reported as right-handed, only 2 each as left and as ambidextrous. Given the small numbers outside the RH group, we could not usefully assess any possible impact of this on commencing or learned abilities.

Participant GMSI backgrounds

Prior to assessment, we compared the Gold-MSI background questionnaire responses of our 68 participants with normative data from the sample of 147,633 participants examined in (Müllensiefen et al. 2014). This comparison was aimed to affirm the novice status we assumed the present sample to have. There were significantly lower ratings (p < .001) than the normative data for all six Gold-MSI subscales (Active engagement; Perceptual abilities; Musical Training; Emotions; Singing abilities; General sophistication).

Supplementary Table 1. Comparison of 68 AMME participant Gold-MSI Survey scores with normative data of 147,633 participants via one-sample *t*-tests. *d* is Cohen's *d*.

**Subscale**	***t***	***p***	**Mean difference**	***d***
Active engagement	-17.83	*p* <.001	-16.81	1.84
Perceptual abilities	-8.6	*p* <.001	-7.91	1.02
Musical training	-36.21	*p* <.001	-19.42	2.24
Emotions	-3.67	*p* <.001	-2.49	0.34
Singing abilities	-11.14	*p* <.001	-10.29	1.59
General sophistication	-15.94	*p* <.001	-27.87	1.57

As a secondary comparison, we examined differences in these survey scores between our 68 participants and a subset of older adults aged 65-80, within the above sample of 147,633 participants. These data were supplied to the research team directly from the original set, via personal correspondence, and were reported as *M* and *SD* values only. The sub-sample were predominantly from the UK (3220, or 84.7%), with the remaining participants primarily from other English-speaking countries. The same analytical approach was used as above, with six separate one-sample *t*-tests, and these results are reported in Supplementary Table 2.

Supplementary Table 2. Comparison of 68 AMME participant Gold-MSI Survey scores with normative data of a sub-sample of 3,800 older adult participants via one-sample *t*-tests.

Subscale	*t*	*p*	Mean difference	*d*
Active engagement	-12.62	p <.001	-11.9	1.32
Perceptual abilities	-6.92	p <.001	-6.37	0.8
Musical training	-30.67	p <.001	-16.45	1.92
Emotions	-1.42	p = .161	-0.96	0.13
Singing abilities	-8.66	p <.001	-8	1.22
General sophistication	-11.97	p <.001	-20.93	1.17

Together, both Supplementary Tables indicate that for five of the six subscales our participants produced significantly lower ratings of sophistication than the normative samples. Yet there was no significant difference for the Emotion subscale between the two older adult samples (i.e., between the AMME sample and the sample of 3,800). We conclude that our 68 participants fit the intended description of music novices, although the data suggest that differences in musical sophistication may not necessarily lead to differences in our emotional responses to music. All comparisons are shown visually in Supplementary Figure 1, with Error bars = +/- 1 *SE*.

Supplementary Figure 1. Comparison of 68 AMME older adult participant Gold-MSI Survey scores (dashed grey line) with normative data of 1) the full sample of 147,633 participants (black line) and 2) the sub-sample of 3,800 older adult participants (solid grey line).

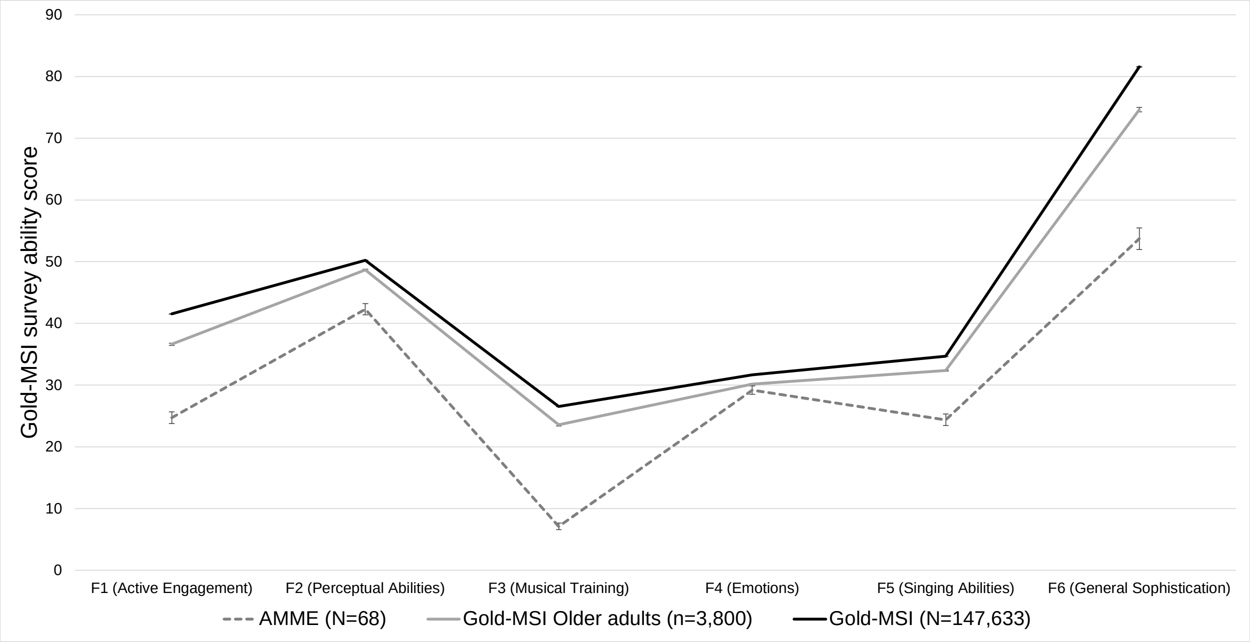

Participant drop-out

The drop-out rate was relatively low, with 13 participants (19.1%) leaving the project at some point across the year. Of the 13 participants who left the study, five of these left in the first 3-month block of learning; the second and third blocks each saw 3 participants leaving, and another two left in the final three months of training. Two participants left at the onset of the COVID-19 pandemic, indicating they did not want to take part in an online study. Various other reasons were given for leaving the program, although many participants indicated this was for reasons outside of their control and expressed a preference to stay within the program. These reasons included changes to working situations, particularly as COVID-19 restrictions were lifted and working flexibility decreased, as well as unexpected changes to personal situations such as increased caring needs. Some participants noted a general lack of time or interest in the program, and one participant left to pursue music education with more contact hours per week, given that additional music study was not possible while taking part in the study. One person participated briefly, but withdrew, objecting to being taught in a prescribed way rather than being able to choose their own path in conjunction with a teacher. This person's data are not included in analyses presented in this paper, apart from those concerning demographics. The low drop-out rate was associated with general enthusiasm for the program (and appreciation of the opportunity to receive free lessons); with notable enjoyment of participating with others, even if online; and also with the relative convenience of online participation in comparison with travelling to our University building, which in the large city of Sydney would have meant a likely journey of at least 15 to 20 km.

Melodies for replication and MIDI file analysis

Participants were invited to propose songs they would like to learn and believed to be generally appealing, and wherever possible this were incorporated into our available set. The resultant set is listed below (somewhat abbreviated names) and examples of the specially composed items (ARC1a, 1b etc ; by author RTD, an active composer/improviser) are also provided. They are mainly popular or traditional songs, with a few classical themes.

ARC1a
ARC1b
ARC2a
ARC2b
ARC3a
ARC3b
Let It Be
Theme based on Beethoven Symphony 5
Jingle Bells
Happy Birthday
Lavenders Blue
Row Row Row Your Boat
Here Comes the Bride
Bye Bye Love

We Wish You a Merry Christmas
Edelweiss
Little Fairy Waltz
Fernando
Where Have All the Flowers Gone
Amazing Grace
Hallelujah
Imagine
Frere Jacques
Hey Jude
Largo
The Lion Sleeps Tonight
London Bridge
Danny Boy
Can You Feel the Love
Mamma Mia
Waltzing Matilda (Chorus)
Michael Row the Boat Ashore
Midnight
Minuet in G
Morning Mood
Blue Moon
Oh What a Beautiful Morning
Silent Night
Ode to Joy
Donna Nobis Pacem
The River is Wide (Water is Wide)
The Rose
Que Sera Sera
On Top of Old Smokey
Somewhere Over the Rainbow
It's Summertime
Sunrise Sunset
Auld Lange Syne
Teach the World to Sing
Love Me Tenderly
Time Goes By
Tura Lura Lural
The Old Walls
Old Waltz
Waterloo
White Christmas
Yellow Submarine
Yesterday

ARC1a has been provided with (Dean et al. 2022) [link] and ARC3a and b are attached. Other files are available on request. These MIDI files are intended for a good grand piano sound, such as Garage Band's Steinway, or originally one from PianoTeq (physical synthesis pianos modelling classic brands).

Analysis of MIDI recordings of performances was done using techniques described in (Dean et al., 2022), and particularly exploited the R dynamic time warping package *dtw*.

Bayesian Statistics and Modelling

The key advantages of Bayesian modelling (van de Schoot et al. 2021) are a full evaluation of the distribution (and not just the point estimate and its variance) of effects, and a capacity to not only test null, but also positive hypotheses. Crudely speaking, it uses the available data much more fully than a frequentist analysis.

The R powerful packages *brms* (Burkner) and *marginal effects* (Bundock) were the main tools, available from RCran (https://cran.r-project.org/). *Brms* (Bayesian regression modelling with Stan) is remarkably flexible, and uses *Stan* as its underlying computational engine for performing in depth Multi Chain Monte Carlo analyses of the data. The package *marginal effects* makes the extraction of the overall effects of individual components of even a complex model with interactions and group effects a much more transparent and practical process, and also allows estimating these under chosen, and when required, semi-idealised (counterfactual), conditions, using predictions from the posterior distribution of the Bayesian model. The posterior is the cumulation of the modelling, representing the maximum information obtained from the data, taking account of any influence of the priors (see below).

A simple R formula in *brms* or elsewhere is of the general form
MDT.ability ~ Session * ctimprep + (Session + ctimprep | PersonID),
using an example from the main text (detailed below). Here the modelled (dependent) variable is on the left of the tilde sign, and the predictors on the right. Ctimprep and Session (==Test Session) have been elaborated in the text, and PersonID (or PID) identifies each individual participant in a coded way. The unbracketed predictors are the population (or main) effects, and the asterisk indicates in interaction i.e. in this case it corresponds to Session + ctimprep + (session x ctimprep). In each bracketed expression (there may be several) are the group (or random) effects, that characterise how each participant (or in some cases each item, melody, stimulus etc) differ in their responses or effects, and which commonly improve models substantially, rendering interpretation clearer and stronger.

Modelling requires establishing a representation of 'priors', that is, evidence-based beliefs, if any, about the parameters of a model. Such belief is progressively updated by the data, and when the data are extensive, and the beliefs expressed conservatively ('uninformative priors'), the priors have no or minute impact. Since we considered there was no strong basis for informative (quantitative) priors in any case here, we standardised relevant measures (so that their distributions for the modelling was mean 0, standard deviation 1), or in some cases scaled them to the range 0 to 1. This then indicates that coefficients even on highly significant and substantially influential parameters in the model should be small, in most cases much less than 1. In turn this permits establishing simple priors that are small values (and this is upheld in our results: see for example, Table 1). We uniformly show results with gaussian priors having

mean 0, sd 1, but other uninformative priors were also tested, with coherent results. If one had previous good evidence of the magnitude of some effect, then it could be set as a prior with a comparable sd; or in an extreme case, even fixed (i.e. not modelled, but predetermined). This was not relevant here. Note that to obtain the Bayesian estimate of the distribution of an outcome variable (as opposed to estimating a model that predicts it optimally), a simple so-called 'descriptive' model can be used, such as MDT.ability ~ Session + (1|PersonID) in the case of the formula introduced above. The optimal predictive model is sometimes termed the 'analytic' model, and we use that terminology in the main text.

Our Bayesian approach included when appropriate a process to combine multiple overlapping measures of a learned feature, that as we mention in the main text is often a desirable way to use some educational measures (e.g. 'creativity', 'divergence' etc). For example, when item 3 of the improvising methods ('change the distance between the notes') is undertaken, one can in theory expect increased diversity and size of sequential pitch *intervals* (especially when considering an upwards and a downwards pair as distinct), but expectations of (non-sequential) pitch diversity per se, or pitch range, are not so clear cut. Hence one may be interested in combining multiple measures, and a strong Bayesian approach to this (which as with Bayesian methods in general, avoids the need for frequentist arbitrary *p* value corrections for multiple analyses) has been developed (Gelman, Hill, and Yajima 2012) and widely used, and it is adopted here when appropriate. It takes proper advantage of the shared (group) variances between people, responses to tunes etc thereby avoiding problems of multiple comparisons and the routine (unprincipled) probability 'corrections' of frequentist approaches. In the model below of Fig 8 in the main text, the final complex group effect term (1 | scoretype : pid), considering a multilevel grouping, is a key feature of Gelman's approach, taking advantage of the shared variance features within both features, scoretype and pid.

Our graphs show the Bayesian Credibility interval (which is simply what it suggests, unlike a frequentist confidence interval), with the median estimate of their centre. The construction and meaning of an evidence ratio (ER) is described in the main text, but it is worth noting here that when an 'infinite' ER is reported, a reasonably conservative estimate of it is the number of data points involved (in other words, it is the maximum that could be achieved given the size of the dataset, not literally infinity).

Additional Results and Model Details

A combined measure of Replication Learning

Given the lack of emphasis on timing training, in developing more combined measures, we chose to use the two pitch measures pitchdtwadj (sequence similarity) and klpitchsim (distributional similarity) described above. In a situation where emphasis was placed on rhythm, timing, metre and the like (unlike our program), the use of the two analogous timing measures should equally be considered. Appendix Analysis Table 3 later shows the complete model. Supplementary Figure 2 below shows the strong result in relation to Training Block Counts (improvisation-replication).

Supplementary Figure 2.

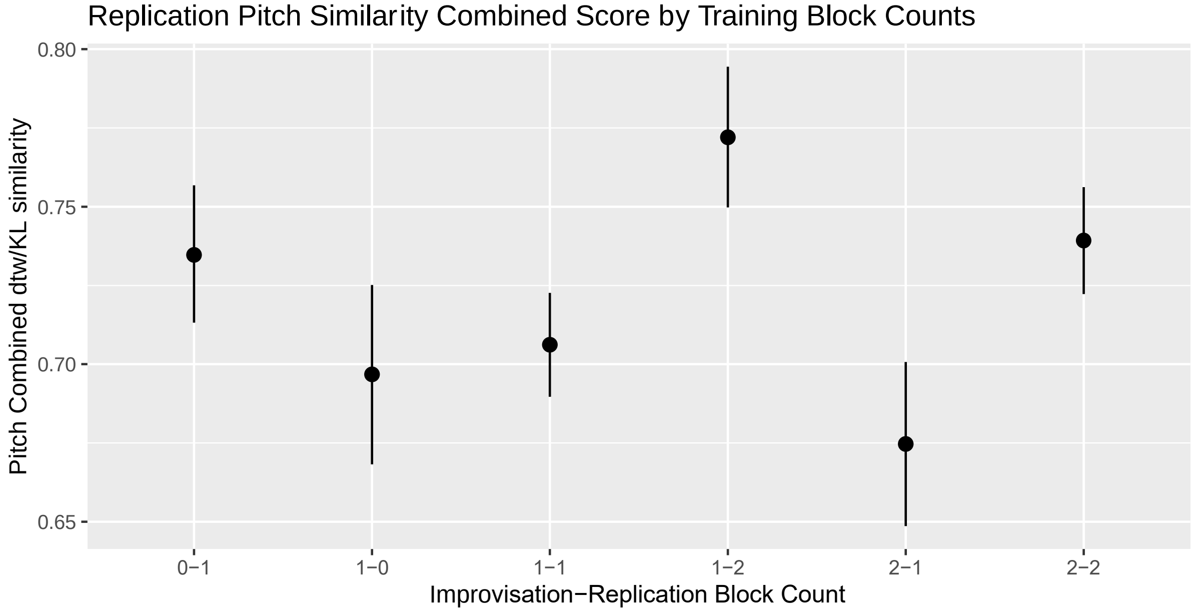

Supplementary Figure 2 Legend. The combined pitch length-adjusted dtw and Kullback-Liebler divergence score vs Improvisation-Replication Block counts.

As noted in the main text, the combined measure showed higher values than the pitch similarity alone, and a pattern very similar to that of main text Figure 4, with the same combination of strong and weak evidence ratios for the differences, and the same relative impacts of replication (positive) and improvisation (somewhat negative). The relative accuracies of the three different Performance Items (not shown), were again completely distinct, and in the same pattern as main text Figure 5. The analysis again confirmed that there was no effect of block (ie time) per se, and that the keyboard blocks produced slightly higher scores (0.736) than the iPad blocks (0.712: evidence ratio strong).

In sum, a successful measure combining pitch sequence accuracy penalised for incomplete performances (few notes), and pitch distribution similarity, confirmed both short- and long-term learning of aural replication skills, and provides an individualisable measure of aural and performance progress that could be used in later analyses, as an alternative or supplement to the use of the MDT.ability, a purely aural musical measure.

Additional Improvisation assessments

The main text emphasises the data limitations resulting from participants' commonly stated choices of employing multiple improvisation methods at once, instead of the 1-2 we recommended. A process of selecting solely the highest of the method-specific scores for each observation (i.e. each performance) was assessed, on the grounds that it might correctly identify the single method to which the participant was giving most attention. This merely flattened the results to uninformative high scores c. 1.5.

Two alternative approaches were assessed to further tease out effects on improvisation of the two musical training modalities by means of the Bayesian models of the large datasets. The first depended on the fact that during the improvisation blocks, most participants prepared some improvisatory techniques for session item 1 (which otherwise, and in all the replication blocks, was a pre-prepared performance of a

specified piece). Thus in these improvisation block cases, Performance item 1 was an ‘applied improvisation’ (in the terminology of (Smith and Dean 1997)), in other words, a piece cultivated over several improvising attempts. Performance Item 1 could be contrasted with Item 4, which was requested to be an improvisation on a short newly chosen note sequence, since both Items 1 and 4 used unspecified methods.

Modelling the performance of session item 1 from the improvisation blocks alone, and using only the 4 pitch scoretypes (2"M1pintervaldivers" 3"M1pintervalrange" 6"M3pitchdivers" 7”M3prange”), given the previously described emphasis on pitch in our teaching, showed a significant positive influence of the first improvisation block, with an evidence ratio (18.95) almost approaching the strong arbitrary cut-off at 19, while the positive influence was retained after the second improvisation block.

For Performance Item 4 (the unspecified and unprepared improvisation) and 5 (the specified but often multiple improvisation methods piece) the same Bayesian approach to analysing the performance of the improvisation tasks was adopted: we used all the relevant available data to obtain a good model of improvisation scores, and then sampled the posterior of that model to extract performance levels respect to ctimprep. For both Performance Item 4 and 5 we reverted to models with separate imprct and repct (improvisation and replication block counts respectively), since there was no 0-0 condition, and since our focus here was on imprct rather than repct or the combined variable ctimprep. In using the posterior distribution to assess the case of Performance item 5, against varying improvisation block counts, the training block count can be set counterfactually to 4 (i.e. maximising number of blocks of training: representing the effect of time/exposure), and repct to 2 (specifying the maximal extent of replication training), nimptasks to 1, but to be compared with 1:7 (all choices). Kbip was set to Keyboard since this showed clearer effects than did the iPad (though there was still higher iPad performance after 2 than 1 or 0 improvisation blocks). Other parameters can be set to any desirable choice or as here to a ‘representative’ value, which sets them at a median or modal value. In the case of session item 4, nimptasks is not relevant (there were none). We again focussed on pitch abilities, using the measures 2, 3, 6, 7, as just mentioned, with the same set of factor levels. For Performance Item 4, the unspecified method improvisation, taking the same communal conditions as just defined, pitch use scores were generally imprct 0 < 2 < 1 but with low evidence ratios (<2.5). In other words, there was no sign of change of any substance with the training. Perhaps this item induced some laxity: without the demand of task focus, participants were possibly playing relatively casually.

The case of Performance Item 5 is discussed in the main text. The model of Fig 8 main text for Item 5 was:

score ~ (kbip + improrep + scoretype + task1 + task2 + task3 + task4 + task5 + task6 + task7 + task8 + task9 + task10 + task11 + task12 + task13 + task14)*imprct + repct*block + (task1 + task2 + task3 + task4 + task5 + task6 + task7 + task8 + task9 + task10 + task11 + task12 + task13 + task14 |scoretype) + (1 | pid) + (1 | scoretype : pid)

Here ‘tasks’ specify the improvisation methods, while kbip indicates whether the completed study block was for keyboard or improvisation training, and similarly improrep indicates whether it was for improvisation or replication training.

So for Fig 8, nimptasks was set at the desirable 1. Gelman’s approach to sharing variance from different scoretypes (here, only the key pitch-relevant scoretypes 2, 3, 6, 7) and pids in a unified score was adopted (see the final group effect in particular, as

introduced above), and a more elaborate set of group effects on task as well as pid included, given the larger extent of data (880 data rows). The model had a sigma of 0.58, and a Bayesian R^2 of 0.569, and thus was good. For other derivative models nimptasks was set to wider ranges, and nimptasks was included as a predictor, given the negative effect of it increasing. See the main text for discussion of the model results.

Learning of Individual Improvisation Tasks

a)Bayesian analysis

The main text describes our approach to Bayesian estimation, and references the Supplementary Table 3.

b) Frequentist analysis of performance of individual improvisation methods

As mentioned, one of the difficulties was that for those participants who undertook the improvisation block first (i.e. as block 1), there is no prior individual baseline measure of their untrained improvisation skills. Given the results noted already indicating a deleterious effect of increasing claimed nimptasks (the number of tasks reported by a participant to be undertaken simultaneously during an improvisation), we reasoned that the best indicator of a successful performance of improvisation method n would occur when it was the sole intended task; however, given the distribution of nimptasks, with most people claiming 2-3 simultaneous tasks, and several claiming more, we also suffered the disadvantage that nimptasks = 1 was a relatively rare occurrence for any given method. Given the descent of task scores below the (standardised) mean of zero when nimptasks reached 4 (Figure 7 in the main text) we chose to analyse scores for a given task when nimptasks = 1, 2 or 3 (but not more). The best 'baseline' comparison in our data might be to compare a performance with nimptasks =1 in which the task was undertaken, with those with nimptasks =1 in which it was not, and similarly for other nimptasks. Commonly the relevant number of data points for such a comparison is limiting. Consequently, we then normally used as baseline a mean value from Performance Session m3 Item 4 (no specific required or declared improvisation tasks), when it followed replication training, for the relevant scoretype (468 observations from 26 participants). We do not report results if there was only a single participant, and the form of the Boxplots to follow indicates whether the data were extensive enough to display the full parameter range of the Boxplot method.

We illustrate the results with some of the more convincing and one of the less convincing patterns that remain positively suggestive. These analyses were necessarily undertaken solely with Performance Item 5 (the only one in which tasks were specified). Note that Performance Item tasks 4 and 8 in particular could both strongly invite reductions or increases in tempo (ioi), and hence our use of an absolute difference measure rather than a raw value (see methods). Also some tasks (e.g., 9/10) are essentially reciprocals of each other, and shared measures. For the following analyses, we prioritised the measure seemingly most specific for the task under study. Of the scoretypes (listed above and in Methods), as previously we completely dismiss 13 in favour of 18, and 14 in favour of 17.

The interpretation of these data, in Supplementary Figure 3 a-e, is discussed in the main text.

Supplementary Table 3. Analysis of Learning of Individual Improvisation Tasks 1-14

			Predicted Standardised scores						
Task: Measure	N samples	Model Bayes R2	imprct 0	1	2	repct 0	1	2	Evidence Ratios >4 and comments
1:8	51	0.49	-0.42	-0.04	-0.24	-.04	0.1	-0.09	
2:9	17	0.75	NA	0.47	0.73	0.75	0.40	0.15	
3:3	30	0.75	0.16	1.44	0.06	0.28	0.18	0.61	Imp 1-0 105.7
4:4	35	0.64	-0.30	0.09	-0.32	-0.13	-0.18	-0.36	Imp 1-0 5.69
5:10	37	0.39	0.18	0.22	0.50	0.29	0.24	0.50	
6:11	5	0.71	NA	-0.13	1.40	-0.13	1.34	1.29	Imp 2-1 9.92. nimptasks had to be set to 3 for predictions
7:12	41	0.85	0.38	-0.36	-0.17	-0.42	0.17	0.15	Rep 1-0 15.88
8:18	31	0.65	-0.12	0.04	0.05	-0.11	0.05	0.15	
9:17	11	0.85	NA	0.16	-0.08	0.32	-0.14	-0.05	nimptasks set to 3
10:17	29	0.41	0.30	-0.16	-0.44	-0.06	-0.21	-0.26	
11:17	2								Cannot be considered
12:15	21	0.56	NA	0.27	0.86	0.44	0.81	0.10	Imp 2-1 5.7. nimptasks set to 3
13:16	15	0.62	-1.09	-0.14	0.01	-0.43	-0.55	-0.17	Imp 2-1 12.18, Imp 3-1 21.12. nimptasks set to 3
14:3	12	0.77	0.19	-0.18	0.01	0.09	-0.05	0.21	

Supplementary Table 3 Legend: Posterior predictions were made with the following *marginal effects* command: predictions(mod, by = "imprct", datagridcf(nimptasks = 1, repct = 0)); and reciprocally for the effects of repct with imprct set at 0. Mod references the model, and datagridcf indicates setting a batch of conditions that may include some not actually realised in the experiment (counterfactual) but within the range of conditions applied. Because the data are limited and predictions have wide Bayesian Credibility Intervals, we indicate any evidence ratios that exceed 4 for a positive effect compared with a specified control level. Whereas a 'strong' evidence ratio of a one-sided effect (>19) indicates at least 95% of the probability mass for the comparison value is in the positive range, a 'weak' value >4 indicates that at least 80% is. In the Scores columns, NA indicates that the prediction command could not provide an estimate (because of a lack in the data). When a zero count value for either imprct or repct is NA, then the baseline for the prediction of the other has to be set at a count of 1 instead of 0. Similarly, in several cases an nimptasks other than 1 had to be chosen given the limited data, as noted in 'comments'.

Supplementary Figure 3 a- e.

Figure 3a

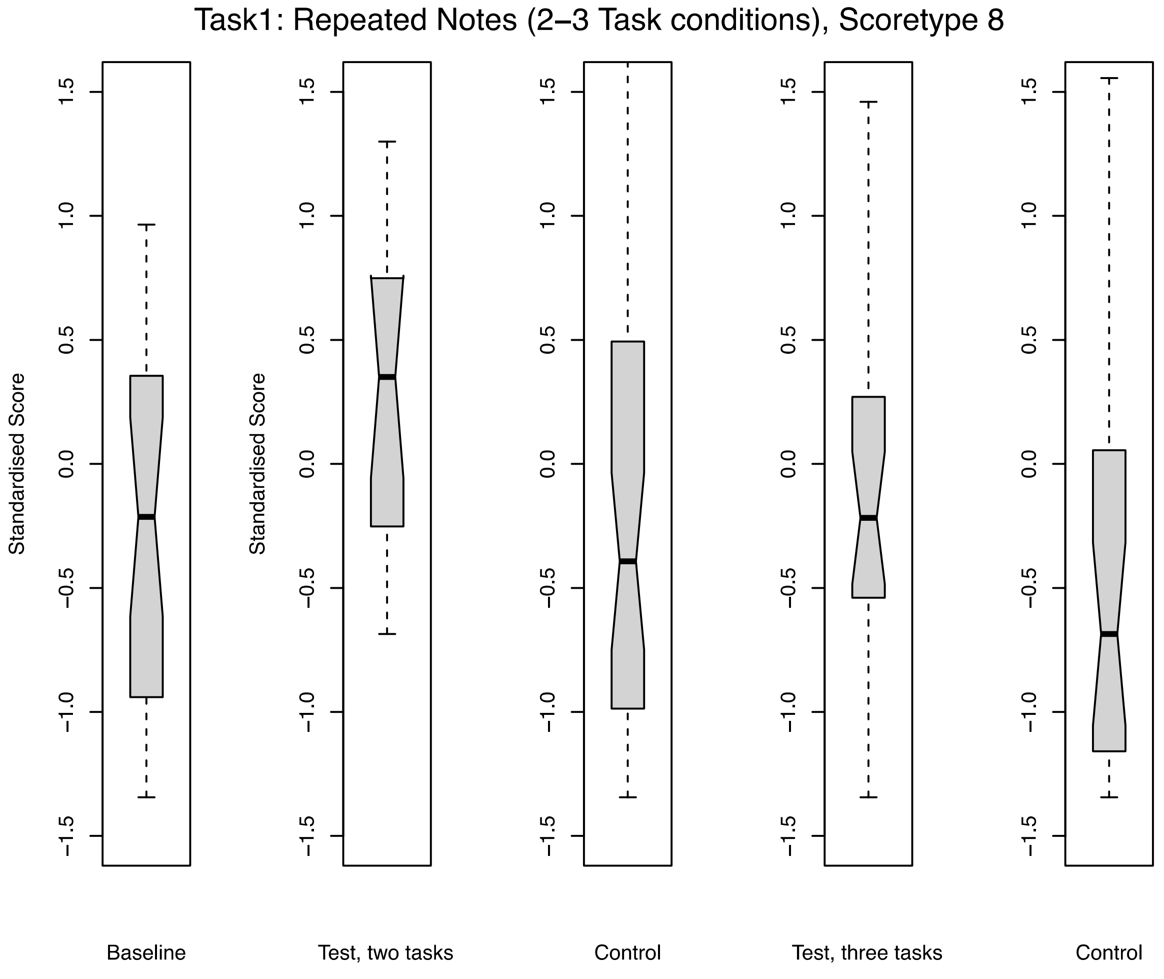

Figure 3b

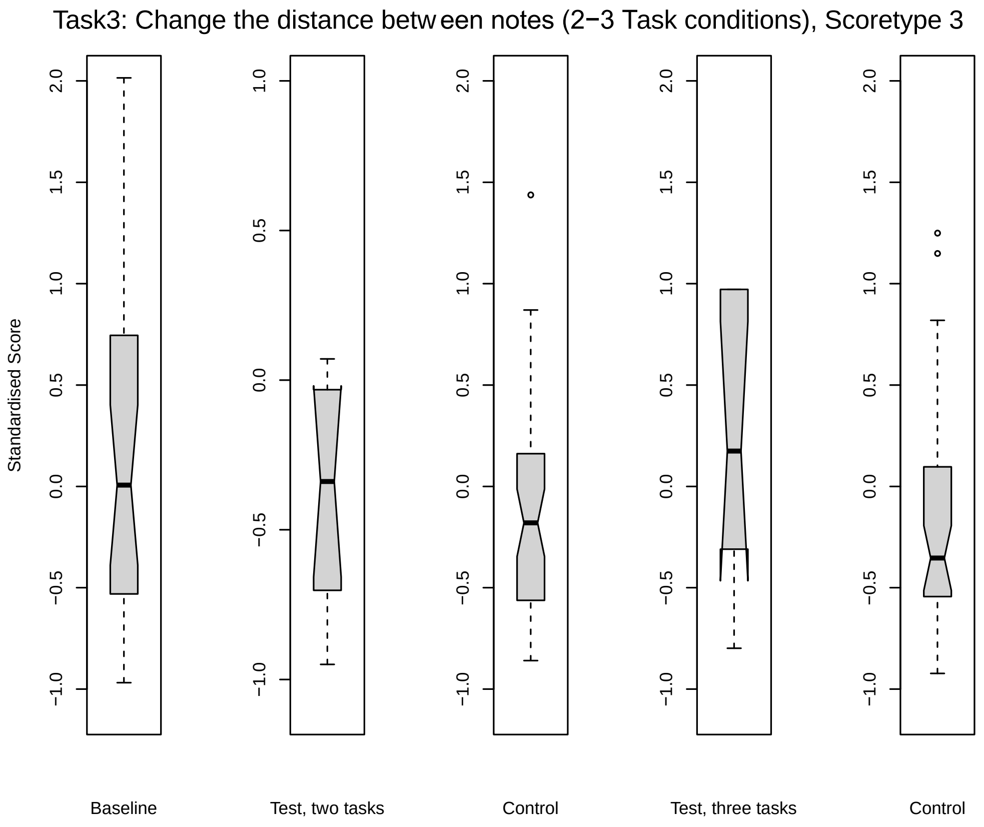

Figure 3c

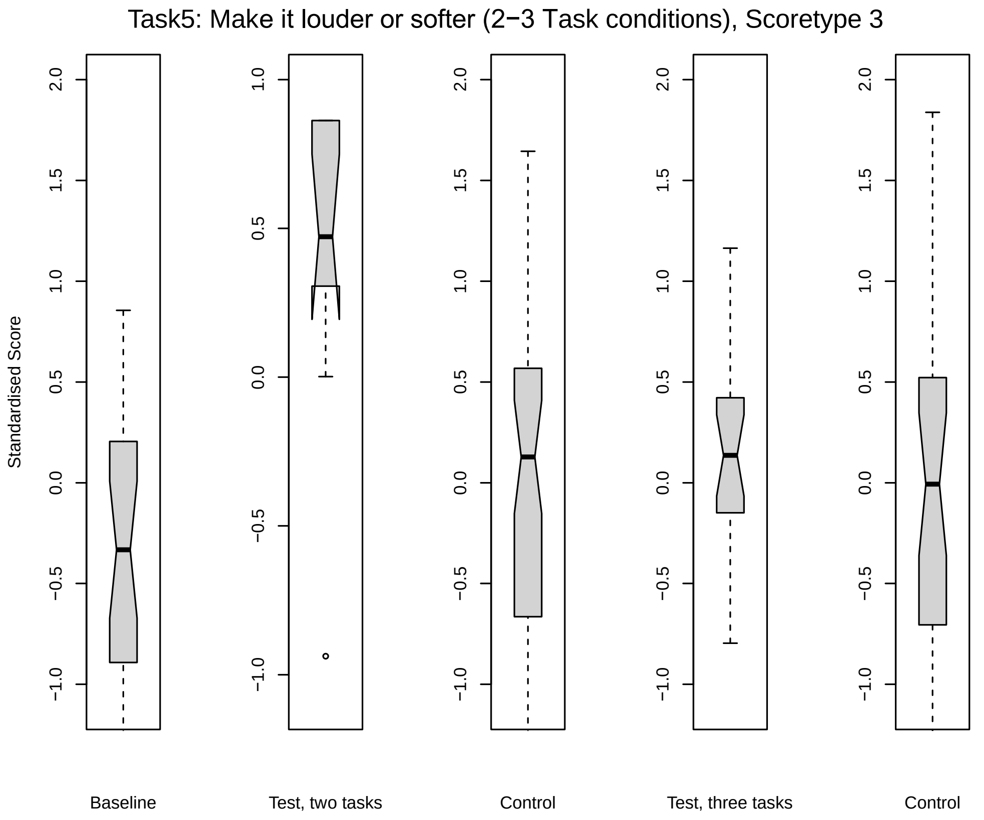

Figure 3d

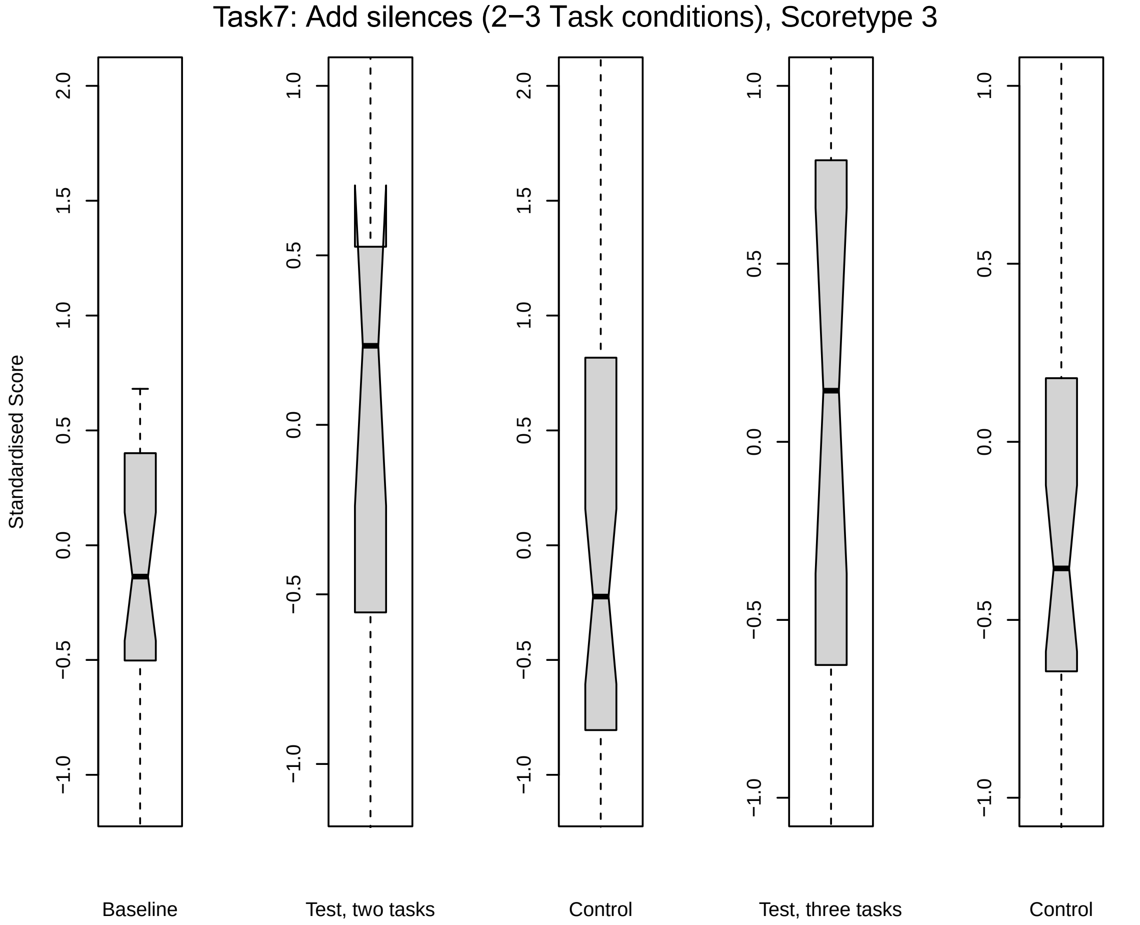

Figure 3e

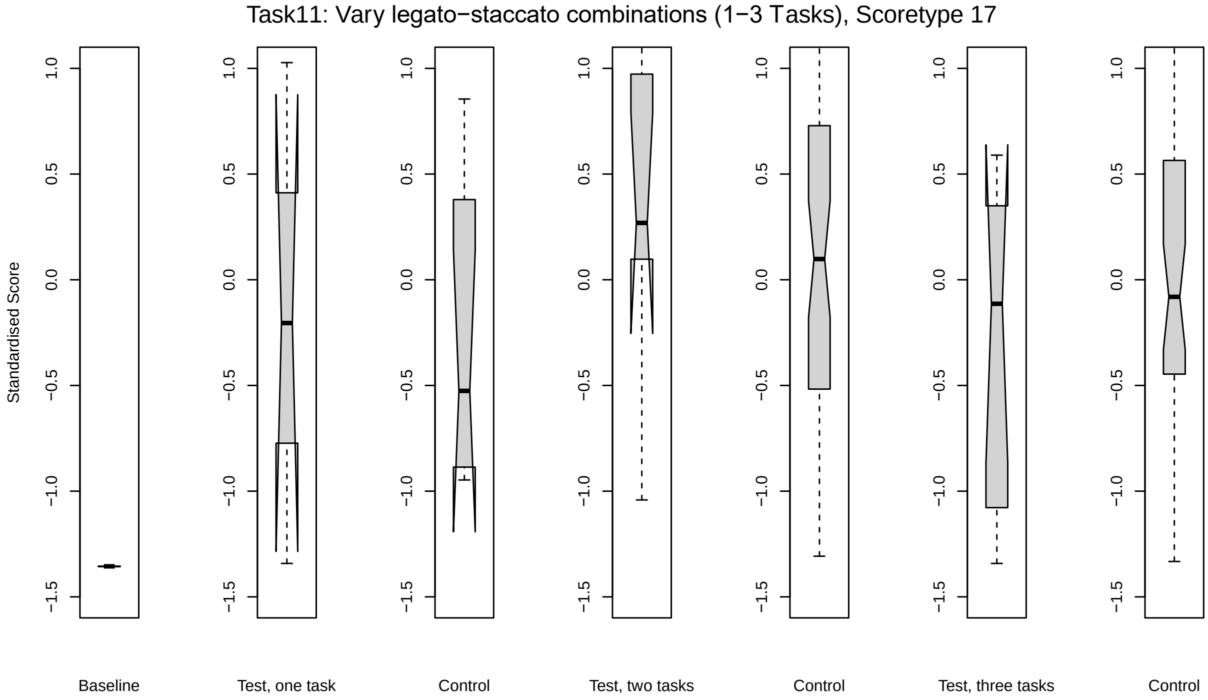

Supplementary Figure 3 a – e. Baseline data are from session item 4 (no specified tasks), while the Test and Control data are from session item 5 where the indicated task was done in the presence of 0-2 other tasks as specified by the participant, and as indicated on the x axis. In each case, 'control' refers to the condition of the Boxplot on its left in terms of number of tasks.

Apart from the session item 'baseline' measure in 3b, Supplementary Figure 3 graphs would all be reasonably indicative of participants' success with the tasks. It needs to be born in mind that the baseline measure is challengeable, and may well be an unnecessarily negative factor.

Overall, we again suggest that success was achieved in several of these tasks. Nevertheless, of the analyses not shown, such Box plots provided no evidence for success in the following methods: 2 (passing notes), 8 (speed changes), 10 (staccato alone: but cf Fig 1e), 12 (retrogrades), 13 (sequences) and 14 (change the set of notes/add some black notes). For some of these, positive evidence in this case was obtained earlier in the Bayesian approach of the main text. The lack of evidence for speed changes may cohere with our lack of teaching emphasis on rhythm; while some of the others (tasks 2,10, 14) may reflect motor difficulties (and the fact than on the iPad with ThumbJam, there is no delineation of 'black' notes from white). The lack of retrogrades was perhaps surprising, given the ease with which they can be made on a keyboard (i.e., the player only needs to keep the fingers in the same location and play them in reverse order), but perhaps they and sequences were still viewed as quite sophisticated methods by our novice participants.

Overall, in bald summary, we consider that these Appendix data support the claim of the main text that most of the improvisation Tasks could be learned within our context and time frame. We anticipate that a focus on any individual task and its concomitant measurement over just a few lessons would normally give a positive outcome.

Self-appraised Keyboard and iPad Fluency with musical tasks

At baseline, as well as in the interview and broad cognitive test sessions following each teaching block and for 6 months after the end of teaching, several questions relevant to musical learning were included. Two dealt with self-appraised efficiency in use of the iPad and the electronic instrument keyboard in musical activity. Supplementary Figure 4 (left) shows the modest improvement in this Bayesian ordinal regression assessment with respect to the use of the iPad. Initial self-appraisal in this case started rather high, with about 20% of people rating their proficiency at the maximum of the Likert scale (7 in this case). The improvement peaked at Test Session m12 (evidence ratio for overall improvement between session 5 and 1 using the link function output was 32, a strong result), after completion of teaching and then remained relatively stable. The response to keyboard training, in which essentially everyone was initially a novice and self-appraised accordingly, was stronger (Supplementary Figure 4 right), with evidence ratios for Test Session m9 and m12 compared with m0 both reported by brms as infinite. In this case too, the enhanced level was retained even at Test Session m18 (6 months after completion of teaching: evidence ratio still infinite). Noting that keyboard performance was in several respects better than that on the iPad — as observed in preceding

analyses — we can probably deduce that participants somewhat confused their feeling of fluency with general uses of an iPad or touchscreen with their specific abilities (less fluent) for playing the ThumbJam keyboard that were relevant here.

Supplementary Figure 4. Self-appraised iPad and Piano playing efficiency by Test Session

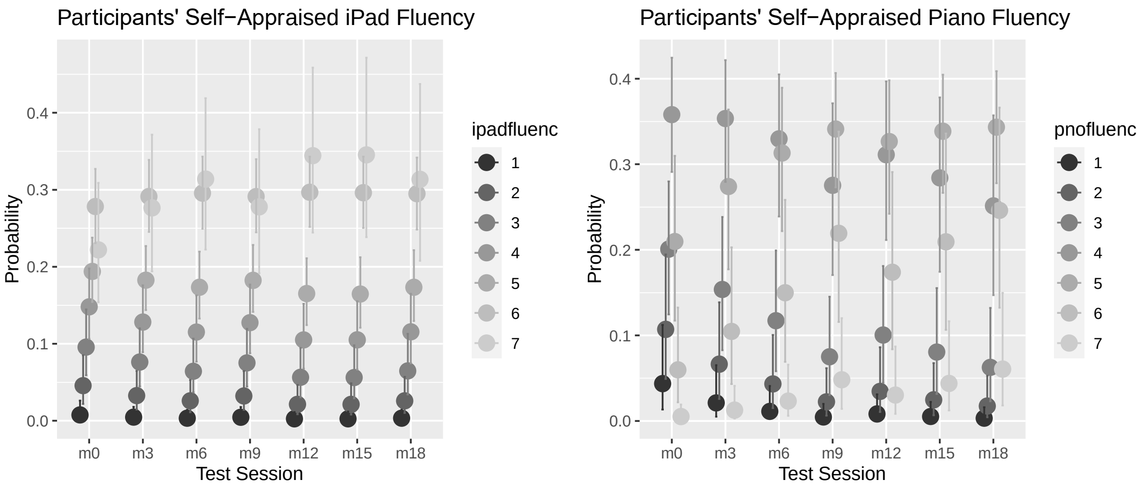

Supplementary Figure 4 Legend: The y axis displays the probability of each of the 7 possible ordinal scores amongst our participants. Note the fairly gradual increase in the probability of the 7 score in the iPad graph, and in the piano graph the enhancement of the 5 score particularly up to session 4 (and retained thereafter) .

Supplementary Figure 5 provides a simplified view of the effects on the linear link function, that is easier to grasp. Now the y axis displays an integrated 'pianofluency' value.

Supplementary Figure 5. Overall linear link values for the 7 sessions of Piano efficiency self-appraisal.

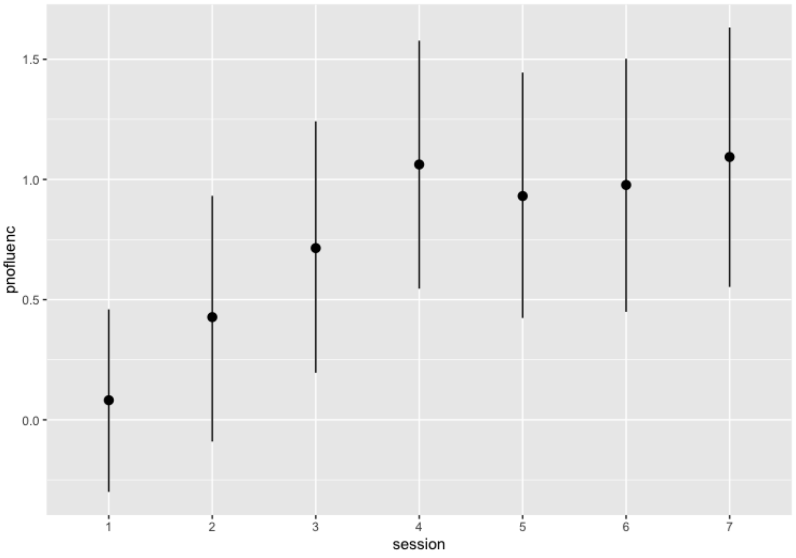

Analysis Models in the Main text
Model 1.
Our developed basic model format, applicable not only to MDT.ability but others also was:
MDT.ability ~ Session * ctimprep + (Session + ctimprep | PersonID)
Session refers to what we call Test Session in the main text.
Separating and Interacting both counts repct and imprct was considered and tested, but was not beneficial (the imprct-session interaction status can be deduced from the repct-session status (or vice versa)). The scale on which MDT.ability is measured ranges from c.-4.4 to + 4.4, and was not further standardised, hence the measured residual sigma (0.53) was reasonable while the Bayesian-R^2 was good at 0.62. Note the importance of using predictors such as Session, ctimprep (or its separated component counts of improvisation and of replication learning blocks) as 'Factors' rather than continuous variables. This allows, for example, Sessions 6 and 7 (post learning), to express the response specific to the stage in question, rather than be included in a single estimated linear coefficient.

Analysis Model 1: Melody distinction test
MDT model
Family: gaussian
Links: mu = identity; sigma = identity
Formula: MDT.ability ~ Session * ctimprep + (Session + ctimprep | PersonID)
Data: MDT_data (Number of observations: 371)
Draws: 4 chains, each with iter = 3000; warmup = 1000; thin = 1;
total post-warmup draws = 8000

Group-Level Effects:
~PersonID (Number of levels: 67)

	Estimate	Est.Error	l-95% CI	u-95% CI	Rhat	Bulk_ESS	Tail_ESS
sd(Intercept)	0.66	0.07	0.52	0.81	1.00	2497	4762
sd(Session2)	0.14	0.11	0.01	0.40	1.00	2655	3052
sd(Session3)	0.19	0.14	0.01	0.49	1.00	1638	3274
sd(Session4)	0.21	0.14	0.01	0.52	1.00	2042	3629
sd(Session5)	0.18	0.12	0.01	0.46	1.00	2490	4020
sd(Session6)	0.34	0.17	0.03	0.67	1.00	1357	1904
sd(Session7)	0.16	0.12	0.01	0.44	1.00	3075	4457
sd(ctimprep0M1)	0.22	0.15	0.01	0.57	1.00	2600	3526
sd(ctimprep1M0)	0.17	0.13	0.01	0.48	1.00	3893	3503
sd(ctimprep1M1)	0.20	0.14	0.01	0.50	1.00	1840	3483
sd(ctimprep1M2)	0.28	0.19	0.01	0.70	1.00	2368	3690
sd(ctimprep2M1)	0.24	0.17	0.01	0.61	1.00	2060	2307
sd(ctimprep2M2)	0.21	0.13	0.01	0.48	1.00	1200	2556
cor(Intercept,Session2)	-0.05	0.27	-0.54	0.46	1.00	11279	6407
cor(Intercept,Session3)	-0.05	0.25	-0.52	0.46	1.00	9318	5768
cor(Session2,Session3)	-0.00	0.26	-0.51	0.50	1.00	6573	5252
cor(Intercept,Session4)	-0.02	0.25	-0.48	0.47	1.00	9560	5440

cor(Session2,Session4)	0.04	0.27	-0.48	0.54	1.00	5777	6170
cor(Session3,Session4)	-0.03	0.26	-0.53	0.48	1.00	5801	5788
cor(Intercept,Session5)	-0.07	0.25	-0.54	0.45	1.00	11296	5425
cor(Session2,Session5)	0.03	0.27	-0.49	0.54	1.00	7059	6346
cor(Session3,Session5)	0.02	0.27	-0.51	0.53	1.00	6847	6768
cor(Session4,Session5)	-0.02	0.27	-0.53	0.51	1.00	7135	6304
cor(Intercept,Session6)	0.01	0.23	-0.43	0.45	1.00	8899	5850
cor(Session2,Session6)	-0.00	0.26	-0.51	0.51	1.00	3868	5094
cor(Session3,Session6)	0.03	0.26	-0.47	0.53	1.00	4235	5593
cor(Session4,Session6)	0.03	0.26	-0.48	0.53	1.00	4296	5654
cor(Session5,Session6)	0.04	0.27	-0.48	0.55	1.00	4179	5879
cor(Intercept,Session7)	0.02	0.25	-0.45	0.49	1.00	11233	5261
cor(Session2,Session7)	0.00	0.27	-0.51	0.52	1.00	7627	6179
cor(Session3,Session7)	0.01	0.26	-0.50	0.51	1.00	8882	6418
cor(Session4,Session7)	0.03	0.26	-0.49	0.53	1.00	8141	6550
cor(Session5,Session7)	0.01	0.27	-0.50	0.53	1.00	6837	6770
cor(Session6,Session7)	0.03	0.27	-0.47	0.53	1.00	7560	6215
cor(Intercept,ctimprep0M1)	-0.07	0.25	-0.54	0.42	1.00	9998	6258
cor(Session2,ctimprep0M1)	-0.03	0.27	-0.54	0.49	1.00	8128	6144
cor(Session3,ctimprep0M1)	-0.01	0.27	-0.53	0.50	1.00	6952	5936
cor(Session4,ctimprep0M1)	0.04	0.27	-0.48	0.54	1.00	6869	6644
cor(Session5,ctimprep0M1)	0.01	0.27	-0.50	0.52	1.00	6780	6436
cor(Session6,ctimprep0M1)	-0.01	0.27	-0.53	0.50	1.00	7362	6810
cor(Session7,ctimprep0M1)	0.01	0.27	-0.51	0.52	1.00	5968	6192
cor(Intercept,ctimprep1M0)	0.00	0.26	-0.50	0.50	1.00	12289	5992
cor(Session2,ctimprep1M0)	-0.04	0.27	-0.55	0.49	1.00	9177	6688
cor(Session3,ctimprep1M0)	0.01	0.26	-0.49	0.52	1.00	9157	6037
cor(Session4,ctimprep1M0)	0.02	0.27	-0.49	0.54	1.00	7742	6690
cor(Session5,ctimprep1M0)	0.03	0.27	-0.50	0.53	1.00	7903	5816
cor(Session6,ctimprep1M0)	0.00	0.27	-0.51	0.52	1.00	7999	6346
cor(Session7,ctimprep1M0)	-0.01	0.26	-0.51	0.51	1.00	6413	6577
cor(ctimprep0M1,ctimprep1M0)	0.01	0.27	-0.52	0.52	1.00	5893	6264
cor(Intercept,ctimprep1M1)	-0.06	0.25	-0.52	0.44	1.00	9479	5608
cor(Session2,ctimprep1M1)	-0.00	0.27	-0.51	0.52	1.00	6994	5739
cor(Session3,ctimprep1M1)	-0.05	0.27	-0.55	0.48	1.00	7220	6339
cor(Session4,ctimprep1M1)	-0.03	0.26	-0.54	0.49	1.00	6368	6108
cor(Session5,ctimprep1M1)	0.03	0.27	-0.50	0.54	1.00	5650	6451
cor(Session6,ctimprep1M1)	0.03	0.26	-0.48	0.53	1.00	6670	5833
cor(Session7,ctimprep1M1)	0.02	0.27	-0.50	0.53	1.00	5909	6437
cor(ctimprep0M1,ctimprep1M1)	-0.01	0.27	-0.52	0.52	1.00	5230	6409
cor(ctimprep1M0,ctimprep1M1)	0.01	0.27	-0.51	0.53	1.00	5178	6466
cor(Intercept,ctimprep1M2)	0.05	0.26	-0.46	0.54	1.00	10902	6230
cor(Session2,ctimprep1M2)	0.03	0.27	-0.50	0.53	1.00	7065	5865
cor(Session3,ctimprep1M2)	-0.02	0.26	-0.53	0.50	1.00	7157	6032
cor(Session4,ctimprep1M2)	-0.04	0.27	-0.54	0.49	1.00	6565	6071
cor(Session5,ctimprep1M2)	0.00	0.27	-0.52	0.51	1.00	6609	6455
cor(Session6,ctimprep1M2)	0.06	0.27	-0.46	0.56	1.00	6512	6119

cor(Session7,ctimprep1M2)	-0.00	0.27	-0.53	0.50	1.00	5813	6429
cor(ctimprep0M1,ctimprep1M2)	0.03	0.27	-0.48	0.55	1.00	5742	6089
cor(ctimprep1M0,ctimprep1M2)	0.00	0.27	-0.52	0.52	1.00	4995	6670
cor(ctimprep1M1,ctimprep1M2)	-0.02	0.26	-0.53	0.49	1.00	5905	6072
cor(Intercept,ctimprep2M1)	-0.07	0.25	-0.55	0.43	1.00	10117	6106
cor(Session2,ctimprep2M1)	0.02	0.27	-0.49	0.53	1.00	7184	6152
cor(Session3,ctimprep2M1)	-0.02	0.26	-0.52	0.49	1.00	7185	6276
cor(Session4,ctimprep2M1)	-0.03	0.27	-0.54	0.49	1.00	7410	6379
cor(Session5,ctimprep2M1)	-0.02	0.27	-0.52	0.50	1.00	6347	6337
cor(Session6,ctimprep2M1)	-0.00	0.26	-0.51	0.51	1.00	6405	6659
cor(Session7,ctimprep2M1)	0.03	0.27	-0.51	0.54	1.00	5447	6407
cor(ctimprep0M1,ctimprep2M1)	0.00	0.27	-0.50	0.51	1.00	5948	6775
cor(ctimprep1M0,ctimprep2M1)	0.02	0.27	-0.50	0.54	1.00	5587	6425
cor(ctimprep1M1,ctimprep2M1)	-0.02	0.27	-0.52	0.50	1.00	5963	6939
cor(ctimprep1M2,ctimprep2M1)	0.00	0.27	-0.51	0.51	1.00	5216	6382
cor(Intercept,ctimprep2M2)	-0.04	0.25	-0.49	0.46	1.00	6999	6031
cor(Session2,ctimprep2M2)	0.04	0.27	-0.49	0.55	1.00	4152	4768
cor(Session3,ctimprep2M2)	0.06	0.27	-0.47	0.58	1.00	3825	5080
cor(Session4,ctimprep2M2)	0.04	0.27	-0.48	0.55	1.00	3803	5222
cor(Session5,ctimprep2M2)	-0.03	0.27	-0.54	0.49	1.00	4696	5532
cor(Session6,ctimprep2M2)	0.05	0.26	-0.46	0.54	1.00	5116	5526
cor(Session7,ctimprep2M2)	-0.04	0.27	-0.54	0.48	1.00	4370	5628
cor(ctimprep0M1,ctimprep2M2)	0.01	0.27	-0.50	0.53	1.00	4641	5946
cor(ctimprep1M0,ctimprep2M2)	0.03	0.26	-0.49	0.53	1.00	4421	6218
cor(ctimprep1M1,ctimprep2M2)	0.06	0.27	-0.46	0.57	1.00	4163	5732
cor(ctimprep1M2,ctimprep2M2)	0.04	0.27	-0.49	0.54	1.00	5074	6260
cor(ctimprep2M1,ctimprep2M2)	0.01	0.26	-0.50	0.51	1.00	5404	6598

Population-Level Effects:

	Estimate	Est.Error	l-95% CI	u-95% CI	Rhat	Bulk_ESS	Tail_ESS
Intercept	-0.71	0.11	-0.92	-0.49	1.00	2279	4348
Session2	0.02	0.35	-0.68	0.70	1.00	6060	5376
Session3	0.04	0.42	-0.78	0.86	1.00	9239	6058
Session4	0.08	0.36	-0.63	0.75	1.00	6925	6148
Session5	0.04	0.38	-0.71	0.79	1.00	7704	6325
Session6	0.03	0.39	-0.73	0.81	1.00	7318	6268
Session7	0.09	0.39	-0.66	0.86	1.00	7224	6041
ctimprep0M1	0.03	0.39	-0.73	0.80	1.00	7411	5780
ctimprep1M0	-0.02	0.40	-0.79	0.77	1.00	7314	6282
ctimprep1M1	0.04	0.41	-0.77	0.84	1.00	8494	6015
ctimprep1M2	0.03	0.40	-0.74	0.82	1.00	7727	5859
ctimprep2M1	0.03	0.40	-0.73	0.83	1.00	7789	5959
ctimprep2M2	0.15	0.32	-0.48	0.80	1.00	6099	6035
Session2:ctimprep0M1	0.04	0.40	-0.74	0.81	1.00	6752	6342
Session3:ctimprep0M1	0.00	0.51	-1.01	1.00	1.00	13815	5833
Session4:ctimprep0M1	0.00	0.51	-0.98	1.00	1.00	14414	5380
Session5:ctimprep0M1	-0.00	0.49	-0.96	0.94	1.00	15557	5812

Session6:ctimprep0M1	-0.00	0.49	-0.96	0.95	1.00	12609	5666
Session7:ctimprep0M1	0.00	0.50	-1.00	1.01	1.00	15661	5805
Session2:ctimprep1M0	-0.03	0.40	-0.81	0.75	1.00	7141	5980
Session3:ctimprep1M0	-0.01	0.51	-0.98	1.00	1.00	13671	5402
Session4:ctimprep1M0	-0.01	0.51	-1.00	0.99	1.00	11673	5625
Session5:ctimprep1M0	0.00	0.50	-0.98	0.98	1.00	13887	6230
Session6:ctimprep1M0	0.01	0.51	-0.98	1.00	1.00	14209	5281
Session7:ctimprep1M0	-0.01	0.48	-0.96	0.91	1.00	12720	5690
Session2:ctimprep1M1	-0.01	0.49	-0.96	0.96	1.00	13433	6115
Session3:ctimprep1M1	0.04	0.40	-0.75	0.83	1.00	8709	6022
Session4:ctimprep1M1	0.01	0.50	-0.97	0.98	1.00	12436	5955
Session5:ctimprep1M1	0.00	0.50	-0.99	0.98	1.00	15588	6082
Session6:ctimprep1M1	-0.00	0.50	-0.98	1.00	1.00	14179	5113
Session7:ctimprep1M1	0.00	0.51	-1.00	1.00	1.00	17004	5146
Session2:ctimprep1M2	-0.00	0.50	-0.97	0.97	1.00	12701	6398
Session3:ctimprep1M2	-0.00	0.50	-0.97	1.00	1.00	15145	5557
Session4:ctimprep1M2	0.03	0.39	-0.73	0.80	1.00	7194	6052
Session5:ctimprep1M2	0.00	0.50	-0.99	0.98	1.00	13893	6217
Session6:ctimprep1M2	0.00	0.51	-0.99	0.99	1.00	15712	5989
Session7:ctimprep1M2	0.00	0.50	-0.98	0.98	1.00	14642	5596
Session2:ctimprep2M1	-0.00	0.50	-0.97	0.98	1.00	13285	5815
Session3:ctimprep2M1	0.00	0.49	-0.95	0.95	1.00	14967	6185
Session4:ctimprep2M1	0.04	0.40	-0.75	0.82	1.00	7678	6120
Session5:ctimprep2M1	-0.00	0.49	-0.96	0.95	1.00	16384	5997
Session6:ctimprep2M1	0.01	0.50	-0.98	0.99	1.00	13606	6268
Session7:ctimprep2M1	-0.00	0.51	-0.99	0.99	1.00	13417	5677
Session2:ctimprep2M2	-0.00	0.49	-0.97	0.97	1.00	13381	5934
Session3:ctimprep2M2	0.00	0.50	-1.00	0.97	1.00	13006	5896
Session4:ctimprep2M2	0.01	0.50	-0.95	0.99	1.00	12300	5832
Session5:ctimprep2M2	0.04	0.39	-0.73	0.83	1.00	8010	5985
Session6:ctimprep2M2	0.02	0.40	-0.76	0.79	1.00	7359	5840
Session7:ctimprep2M2	0.10	0.39	-0.65	0.88	1.00	7157	5689

Family Specific Parameters:

	Estimate	Est.Error	l-95% CI	u-95% CI	Rhat	Bulk_ESS	Tail_ESS
sigma	0.53	0.03	0.47	0.59	1.00	1733	3227

BayesianR2:

	Estimate	Est.Error	Q2.5	Q97.5
R2	0.6232653	0.03762277	0.5474623	0.6963111

Note: All but MDT.ability used as factors.

Analysis Model 2: Pitchdtwladj replication performance.

Family: gaussian
Links: mu = identity; sigma = identity
Formula: score ~ sessitem * ctimprep + kbip + (1 | tune) + (ctimprep | pid) + (1 | group)
Data: data7 (Number of observations: 523)

Draws: 4 chains, each with iter = 3000; warmup = 1000; thin = 1;
total post-warmup draws = 8000

Group-Level Effects:

~group (Number of levels: 10)

	Estimate	Est.Error	l-95% CI	u-95% CI	Rhat	Bulk_ESS	Tail_ESS
sd(Intercept)	0.05	0.03	0.00	0.12	1.00	1573	2196

~pid (Number of levels: 62)

	Estimate	Est.Error	l-95% CI	u-95% CI	Rhat	Bulk_ESS	Tail_ESS
sd(Intercept)	0.11	0.02	0.07	0.14	1.00	2722	4673
sd(ctimprep1M0)	0.05	0.03	0.00	0.13	1.00	4018	4266
sd(ctimprep1M1)	0.05	0.03	0.00	0.12	1.00	1239	3264
sd(ctimprep1M2)	0.05	0.03	0.00	0.12	1.00	2993	3696
sd(ctimprep2M1)	0.08	0.05	0.00	0.18	1.00	2114	2612
sd(ctimprep2M2)	0.03	0.02	0.00	0.08	1.00	3302	3834
cor(Intercept,ctimprep1M0)	0.03	0.36	-0.68	0.71	1.00	9597	5001
cor(Intercept,ctimprep1M1)	-0.16	0.34	-0.73	0.56	1.00	5902	5652
cor(ctimprep1M0,ctimprep1M1)	-0.01	0.38	-0.71	0.71	1.00	4838	5481
cor(Intercept,ctimprep1M2)	-0.04	0.36	-0.70	0.64	1.00	8078	5994
cor(ctimprep1M0,ctimprep1M2)	0.02	0.38	-0.70	0.72	1.00	5723	6338
cor(ctimprep1M1,ctimprep1M2)	0.03	0.37	-0.69	0.72	1.00	6613	6096
cor(Intercept,ctimprep2M1)	0.18	0.33	-0.48	0.76	1.00	5606	5699
cor(ctimprep1M0,ctimprep2M1)	0.07	0.38	-0.65	0.75	1.00	4575	5166
cor(ctimprep1M1,ctimprep2M1)	0.03	0.38	-0.70	0.73	1.00	4519	5832
cor(ctimprep1M2,ctimprep2M1)	-0.00	0.37	-0.71	0.71	1.00	4983	6380
cor(Intercept,ctimprep2M2)	-0.00	0.37	-0.69	0.69	1.00	8997	5552
cor(ctimprep1M0,ctimprep2M2)	0.05	0.39	-0.68	0.75	1.00	7382	5687
cor(ctimprep1M1,ctimprep2M2)	0.03	0.38	-0.70	0.73	1.00	7255	6231
cor(ctimprep1M2,ctimprep2M2)	0.05	0.38	-0.67	0.73	1.00	6230	6772
cor(ctimprep2M1,ctimprep2M2)	0.01	0.38	-0.69	0.72	1.00	6305	7073

~tune (Number of levels: 43)

	Estimate	Est.Error	l-95% CI	u-95% CI	Rhat	Bulk_ESS	Tail_ESS
sd(Intercept)	0.15	0.03	0.10	0.21	1.00	2158	4419

Population-Level Effects:

	Estimate	Est.Error	l-95% CI	u-95% CI	Rhat	Bulk_ESS	Tail_ESS
Intercept	0.69	0.06	0.57	0.81	1.00	2783	4101
sessitem2	-0.37	0.07	-0.51	-0.23	1.00	2268	3648
sessitem3	-0.24	0.07	-0.38	-0.10	1.00	2433	3832
ctimprep1M0	0.16	0.21	-0.25	0.58	1.00	3534	5031
ctimprep1M1	0.03	0.07	-0.10	0.17	1.00	2613	4176
ctimprep1M2	0.11	0.06	-0.01	0.24	1.00	2910	4618
ctimprep2M1	0.00	0.57	-1.10	1.11	1.00	4413	4792
ctimprep2M2	-0.06	0.07	-0.20	0.07	1.00	2596	4317
kbipK	0.04	0.02	-0.00	0.08	1.00	8385	6248

sessitem2:ctimprep1M0 -0.17 0.22 -0.60 0.25 1.00 3663 4703
sessitem3:ctimprep1M0 -0.21 0.22 -0.62 0.22 1.00 3599 4944
sessitem2:ctimprep1M1 -0.03 0.08 -0.19 0.12 1.00 2645 4160
sessitem3:ctimprep1M1 -0.05 0.08 -0.21 0.11 1.00 2848 4237
sessitem2:ctimprep1M2 0.00 0.08 -0.16 0.17 1.00 2969 4383
sessitem3:ctimprep1M2 -0.09 0.08 -0.26 0.08 1.00 3290 4740
sessitem2:ctimprep2M1 0.00 0.57 -1.10 1.12 1.00 4442 4976
sessitem3:ctimprep2M1 0.03 0.58 -1.07 1.15 1.00 4399 4770
sessitem2:ctimprep2M2 0.10 0.08 -0.07 0.25 1.00 2675 4323
sessitem3:ctimprep2M2 0.11 0.08 -0.05 0.26 1.00 2697 4400

Family Specific Parameters:
Estimate Est.Error l-95% CI u-95% CI Rhat Bulk_ESS Tail_ESS
sigma 0.19 0.01 0.18 0.20 1.00 5087 5737
Bayesian R2:
Estimate Est.Error Q2.5 Q97.5
R2 0.5348164 0.02628418 0.4808505 0.5833413

Performance Item (labelled sessitem) as above is 1,2, or 3 defining the pre-prepared piece (1), the newly attempted piece (2) and then the latter item briefly auditioned and practised (3). repct is a count of the number of blocks of replication training a participant has experienced (0-2), while (training) block correspondingly ranges from 1-4. Kbip distinguishes training on the electronic keyboard (K) vs the iPad, while improrep distinguishes whether the current block was one of improvisation or replication (R) study. Ctimprec shows the counts of the number of improvisation and replication blocks a participant has experienced in the format impMrep while group designates the 10 different groups that were taught. Tune is the piece being played, and participants are identified individually by pid. The model has respectably low residual variance (sigma), and its Bayesian R2 is also fair. The model was designed to be broadly inclusive, and no model was more predictive. All but score (continuous numeric) were used as factors.

Analysis Model 3:
A composite pitch performance measure
Family: gaussian
Links: mu = identity; sigma = identity
Formula: score ~ sessitem * ctimprep * scoretype + kbip + group + (1 + scoretype | tune) + (1 | scoretype:pid) + (1 | group)
Data: data57 (Number of observations: 1046)
Draws: 4 chains, each with iter = 3000; warmup = 1000; thin = 1;
total post-warmup draws = 8000

Group-Level Effects:
~group (Number of levels: 10)
Estimate Est.Error l-95% CI u-95% CI Rhat Bulk_ESS Tail_ESS
sd(Intercept) 0.13 0.12 0.00 0.43 1.00 1618 1915

~scoretype:pid (Number of levels: 124)

	Estimate	Est.Error	l-95% CI	u-95% CI	Rhat	Bulk_ESS	Tail_ESS
sd(Intercept)	0.08	0.01	0.06	0.09	1.00	3547	4724

~tune (Number of levels: 43)

	Estimate	Est.Error	l-95% CI	u-95% CI	Rhat	Bulk_ESS	Tail_ESS
sd(Intercept)	0.04	0.01	0.02	0.06	1.00	3750	3696
sd(scoretype7)	0.14	0.02	0.10	0.19	1.00	3716	5535
cor(Intercept,scoretype7)	0.87	0.14	0.50	1.00	1.00	1659	2101

Population-Level Effects:

	Estimate	Est.Error	l-95% CI	u-95% CI	Rhat	Bulk_ESS	Tail_ESS
Intercept	0.94	0.11	0.72	1.19	1.00	2759	3113
sessitem2	-0.09	0.04	-0.17	-0.01	1.00	2841	5022
sessitem3	-0.06	0.04	-0.14	0.01	1.00	2587	4548
ctimprep1M0	0.01	0.13	-0.24	0.27	1.00	4269	5205
ctimprep1M1	-0.01	0.04	-0.10	0.07	1.00	3296	5052
ctimprep1M2	0.02	0.04	-0.05	0.10	1.00	3308	4693
ctimprep2M1	-0.00	0.29	-0.56	0.58	1.00	6202	5911
ctimprep2M2	-0.04	0.04	-0.12	0.04	1.00	3233	4958
scoretype7	-0.25	0.05	-0.35	-0.15	1.00	2846	5016
kbipK	0.03	0.01	0.01	0.05	1.00	17723	6451
group10	0.03	0.17	-0.35	0.39	1.00	5876	4442
group2	-0.02	0.17	-0.40	0.35	1.00	4321	3748
group3	0.04	0.18	-0.35	0.42	1.00	5209	4074
group4	0.03	0.18	-0.36	0.40	1.00	4932	4021
group5	0.03	0.18	-0.36	0.40	1.00	4730	3315
group6	-0.02	0.17	-0.40	0.34	1.00	4715	3913
group7	0.10	0.18	-0.29	0.47	1.00	4679	3794
group8	0.04	0.18	-0.36	0.41	1.00	4609	3490
group9	0.05	0.18	-0.34	0.40	1.00	3770	3442
sessitem2:ctimprep1M0	-0.03	0.13	-0.28	0.23	1.00	4345	5596
sessitem3:ctimprep1M0	-0.02	0.13	-0.28	0.24	1.00	4320	5363
sessitem2:ctimprep1M1	0.01	0.05	-0.09	0.11	1.00	3273	4741
sessitem3:ctimprep1M1	0.02	0.05	-0.09	0.12	1.00	3144	4600
sessitem2:ctimprep1M2	-0.01	0.05	-0.11	0.11	1.00	3458	5773
sessitem3:ctimprep1M2	0.01	0.05	-0.10	0.11	1.00	3175	5517
sessitem2:ctimprep2M1	-0.01	0.29	-0.59	0.55	1.00	6130	6125
sessitem3:ctimprep2M1	0.01	0.29	-0.57	0.57	1.00	6230	5977
sessitem2:ctimprep2M2	0.04	0.05	-0.07	0.14	1.00	3254	4846
sessitem3:ctimprep2M2	0.05	0.05	-0.05	0.15	1.00	3035	4345
sessitem2:scoretype7	-0.31	0.06	-0.44	-0.19	1.00	3163	5077
sessitem3:scoretype7	-0.20	0.06	-0.33	-0.08	1.00	2934	5166
ctimprep1M0:scoretype7	0.13	0.17	-0.21	0.46	1.00	4598	5112
ctimprep1M1:scoretype7	0.04	0.06	-0.08	0.16	1.00	3512	4772
ctimprep1M2:scoretype7	0.08	0.06	-0.03	0.20	1.00	3375	5211

ctimprep2M1:scoretype7	0.01	0.29	-0.56	0.58	1.00	6798	5029
ctimprep2M2:scoretype7	-0.05	0.06	-0.17	0.07	1.00	3178	4937
sessitem2:ctimprep1M0:scoretype7	-0.13	0.18	-0.49	0.20	1.00	4678	4121
sessitem3:ctimprep1M0:scoretype7	-0.18	0.18	-0.52	0.17	1.00	4535	4559
sessitem2:ctimprep1M1:scoretype7	-0.04	0.07	-0.18	0.11	1.00	3489	5502
sessitem3:ctimprep1M1:scoretype7	-0.06	0.07	-0.21	0.08	1.00	3563	5302
sessitem2:ctimprep1M2:scoretype7	0.01	0.08	-0.15	0.16	1.00	3696	5529
sessitem3:ctimprep1M2:scoretype7	-0.10	0.08	-0.25	0.06	1.00	3527	5296
sessitem2:ctimprep2M1:scoretype7	0.01	0.29	-0.56	0.58	1.00	6586	4892
sessitem3:ctimprep2M1:scoretype7	0.01	0.29	-0.56	0.58	1.00	6629	5268
sessitem2:ctimprep2M2:scoretype7	0.08	0.07	-0.07	0.22	1.00	3249	4794
sessitem3:ctimprep2M2:scoretype7	0.07	0.07	-0.07	0.22	1.00	3307	4820

Family Specific Parameters:

	Estimate	Est.Error	l-95% CI	u-95% CI	Rhat	Bulk_ESS	Tail_ESS
sigma	0.14	0.00	0.13	0.15	1.00	9487	6614

Bayesian-R2 0.77.

Note: again only score is used as a continuous variable.

References

Dean, Roger T, Chmiel, Anthony, Radnan, Madeleine, Taylor, John R, and MacRitchie, Jennifer. 2022. "AMMRI: a computational assessment tool for music novices' replication and improvisation tasks." *Journal Of New Music Research* 51 (4-5):262-277. https://doi.org/10.1080/09298215.2023.2270973

Gelman, Andrew, Jennifer Hill, and Masanao Yajima. 2012. "Why we (usually) don't have to worry about multiple comparisons." *Journal of research on educational effectiveness* 5 (2):189-211.

Müllensiefen, Daniel, Bruno Gingras, Jason Musil, and Lauren Stewart. 2014. "The musicality of non-musicians: An index for assessing musical sophistication in the general population." *PLoS One* 9 (2):e89642.

Smith, Hazel, and Roger T. Dean. 1997. *Improvisation, Hypermedia and the Arts since 1945*. London: Routledge.

van de Schoot, Rens, Sarah Depaoli, Ruth King, Bianca Kramer, Kaspar Märtens, Mahlet G Tadesse, Marina Vannucci, Andrew Gelman, Duco Veen, and Joukje Willemsen. 2021. "Bayesian statistics and modelling." *Nature Reviews Methods Primers* 1 (1):1-26.
